# A New Basal Hadrosauroid Dinosaur from the Lower Cretaceous Khok Kruat Formation in Nakhon Ratchasima Province, Northeastern Thailand

**DOI:** 10.1371/journal.pone.0145904

**Published:** 2015-12-30

**Authors:** Masateru Shibata, Pratueng Jintasakul, Yoichi Azuma, Hai-Lu You

**Affiliations:** 1 Institute of Dinosaur Research, Fukui Prefectural University, 4–1–1 Kenjojima, Matsuoka, Eiheiji–Cho, Fukui, 910–1195, Japan; 2 Fukui Prefectural Dinosaur Museum, 51–11 Muroko, Terao, Katsuyama, Fukui, 911–8601, Japan; 3 Northeastern Research Institute of Petrified Wood and Mineral Resources, Nakhon Ratchasima Rajabhat University, 184 Moo 7 Suranaree Subdistict, Mueang, Nakhon Ratchasima District, Nakhon Ratchasima, 30000, Thailand; 4 Key Laboratory of Vertebrate Evolution and Human Origins of Chinese Academy of Sciences, Institute of Vertebrate Paleontology and Paleoanthropology, Chinese Academy of Sciences, Beijing, 100044, PR China; Institute of Vertebrate Paleontology and Paleoanthropology Chinese Academy of Sciences, CHINA

## Abstract

A new basal hadrosauroid dinosaur from the Lower Cretaceous Khok Kruat Formation of Thailand, *Sirindhorna khoratensis* gen. et sp. nov is described. The new taxon is based on composite skull and mandible including premaxilla, maxilla, jugal, quadrate, braincases, predentary, dentaries, surangular, and maxillary and dentary teeth. It is diagnostic by such characters as, sagittal crest extending along entire dorsal surface of the parietal and reaching the frontoparietal suture (autapomorphy), transversely straight frontoparietal suture, caudodorsally faced supraoccipital, no participation of the supraoccipital in the foramen magnum, mesiodistally wide leaf-shaped dentary tooth with primary and secondary ridges on the lingual surface of the crown, perpendicularly-erected and large coronoid process of dentary, and nonvisible antorbital fossa of the maxilla in lateral view. Phylogenetic analysis revealed *S*. *khoratensis* as among the most basal hadrosauroids. *Sirindhorna khoratensis* is the best-preserved iguanodontian ornithopod in Southeast Asia and sheds new light to resolve the evolution of basal hadrosauriforms.

## Introduction

Fossil records of non-hadrosaurid hadrosauriform dinosaurs in Asia have been accumulated in this century [1–11]. Although these discoveries mainly came from China and Mongolia, new findings have been known from Uzbekistan [12] Kazakhstan[13], Japan [14,15] and Thailand [16,17]. However, well-preserved iguanodontian specimens were restricted in China and Mongolia; for instance, *Jinzhousaurus yangi* was known as the almost complete articulated skeleton found from Liaoning Province [6], *Xuwulong yueluni* was represented by an articulated skeleton without appendages from Gansu Province of China [11], and *Probactrosaurus gobiensis* from Inner Mongolia was described including several individuals of cranium and post cranial portions [5]. In contrast, although two iguanodontians known from the Lower Cretaceous of Thailand, *Siamodon* (maxilla and referred braincase; [16]) and *Ratchasimasaurus* (dentary; [17]), none of them provides enough characters to discuss their phylogenies in detail. The new taxon in this study is known from extensive remains including a disarticulated skull and mandibles, and is much more complete than material of the aforementioned Thailand iguanodontians. This new material was collected from one locality of the Lower Cretaceous Khok Kruat Formation during the first term of Japan-Thailand Dinosaur Project (abbreviated as JTDP), including the preliminary excavation by NRRU in 2005. We describe this material and discuss its phylogenetic position based on a cladistic analysis.

### Geological setting

**Fig 1:**
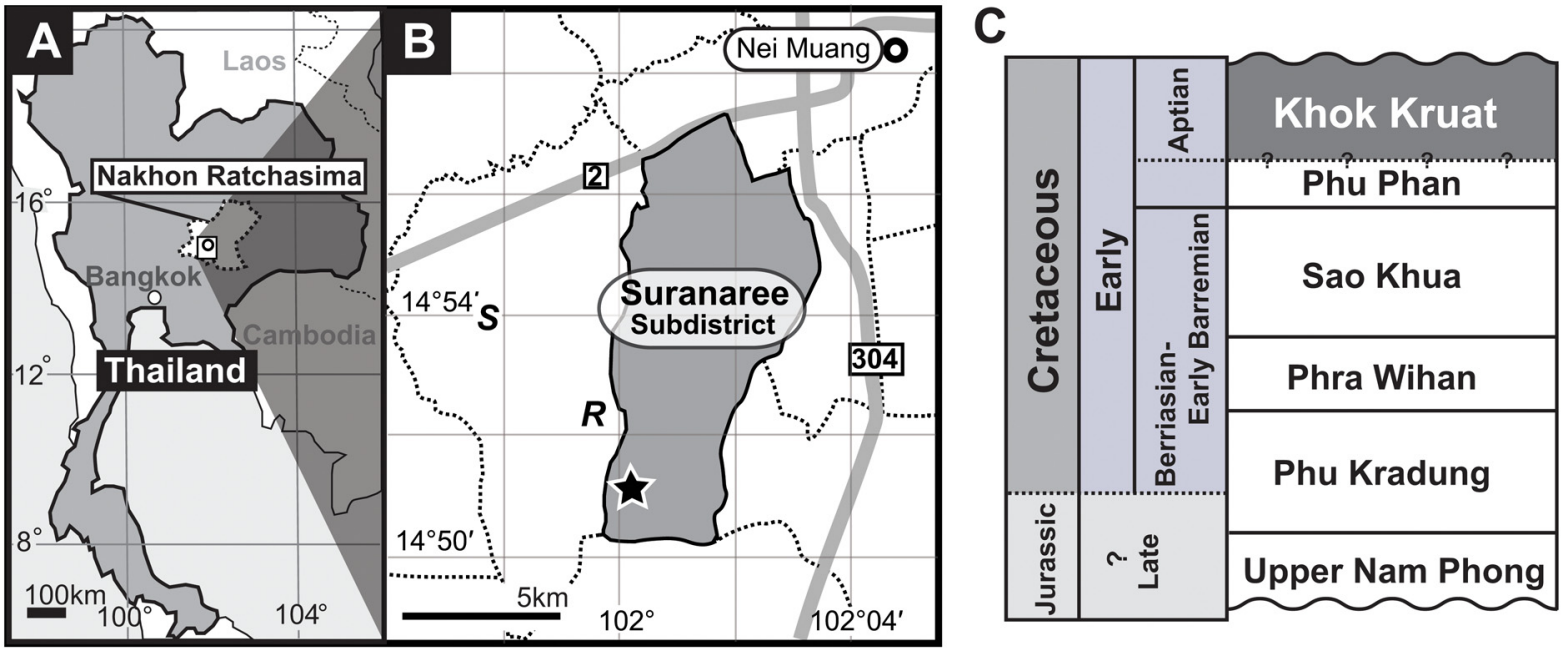
Locality map and stratigraphic column for *Sirindhorna*. (A) Map of Nakhon Ratchasima Province, Thailand, (B) localities of *Sirindhorna* (star mark), *Ratchasimasuarus* (*R*) and *Siamodon* (*S*), (C) stratigraphic column for the Khorat Group (after [19]).

Since 2007, the collaborative research project (JTDP) between Fukui Prefectural Dinosaur Museum (FPDM), Japan and the Northeastern Research Institute of Petrified Woods and Mineral Resources, Nakhon Ratchasima Rajabhat University (NRRU), Thailand, have uncovered abundant vertebrate fossils from a site in the Lower Cretaceous Khok Kruat Formation in Suranaree Subdistrict, Muaeng Nakhon Ratchasima District, Nakhon Ratchasima (Fig 1). The Khok Kruat Formation is the uppermost unit of the Khorat Group and is distributed widely in the Khorat Basin of northeastern Thailand [18]. This formation consists of siltstone, mudstone, sandstone and conglomerate including calcareous nodules, and was deposited in the meandering river under the semi-arid to arid environment [18–20]. The precise age of this formation has not been determined yet due to lack of diagnostic index fossils to date. However, no direct information from the Khok Kruat Formation was obtained to corroborate this age in [21]. Traditionally, an Aptian age is accepted for this formation based on palynological data, the occurrences of the fresh water hybodont shark *Thaiodus ruchae* and the basal ceratopsian *Psittacosaurus sattayaraki*, and the age of the overlying Maha Sarakham Formation (Albian–Cenomanian) [20, 22–24]. We follow the Aptian age determination in this study (Fig 1C).

The Khok Kruat Formation usually does not crop out in Nakhon Ratchasima and thin reddish soil usually covers over those rocks. In our excavation site, for instance, corns and tapiocas are normally planted except for our excavation period. For that reason, local farmers discovered vertebrate remains in this locality when they dug to make a small reservoir in the area prior to the start of this project. This situation made it difficult to locate the bonebed horizon and trace the distribution of fossils at the site. In our project, we identified the bonebed and collected not only fossils but also taphonomic information. Geology, taphonomy and other researches on this excavation site will be done in separate papers.

The bonebed is the reddish conglomeratic sandstone with rich calcareous nodules. Variable vertebrate remains had been unearthed from this horizon as well as other sites of the Khok Kruat Formation [23]; i.e., bony fish scales, hybodont shark teeth, crocodilyforms and dinosaurs. No identifiable invertebrate and plant fossils have been recognized in this horizon.

## Nomenclatural Acts

The electronic edition of this article conforms to the requirements of the amended International Code of Zoological Nomenclature, and hence the new names contained herein are available under that Code from the electronic edition of this article. This published work and the nomenclatural acts it contains have been registered in ZooBank, the online registration system for the ICZN. The ZooBank LSIDs (Life Science Identifiers) can be resolved and the associated information viewed through any standard web browser by appending the LSID to the prefix “http://zoobank.org/”. The LSID for this publication is: urn:lsid:zoobank.org:pub:DA950492-43CB-4964-ACB7-B06AA227BAB0. The electronic edition of this work was published in a journal with an ISSN, and has been archived and is available from the following digital repositories: PubMed Central, LOCKSS.

## Materials and Methods

The specimens described here (NRRU3001-7, 14, 28, 65,137,157, 163,166, 167, 169, 175, 179, A1956, A2035, A2047, A3623, A3630, A3649, Northeastern Research Institute of Petrified Wood and Mineral Resources, Nakhon Ratchasima Rajabhat University, Thailand) are permanently reposited in the collection of the Northeastern Research Institute of Petrified Wood and Mineral Resources, Nakhon Ratchasima Rajabhat University, Thailand and are accessible to all researchers. No permits were required for the described study, which complied with all relevant regulations. The excavation and collection of fossil remains were agreed with the landowner and officially reported to the Department of the Mineral Resources, Thailand.

## Systematic Paleontology

Dinosauria Owen, 1842 [25]

Ornithischia Seeley, 1887 [26]

Iguanodontia Dollo, 1888 [27] *sensu* Sereno, 2005 [28]

Ankylopollexia Sereno, 1986 [29] *sensu* Sereno, 2005 [28]

Styracosterna Sereno, 1986 [29] *sensu* Sereno, 2005 [28]

Hadrosauriformes Sereno, 1997 [30] *sensu* Sereno, 1998 [31]

Hadrosauroidea Sereno, 1986 [29] *sensu* Sereno, 2005 [28]

*Sirindhorna* gen. nov.

urn:lsid:zoobank.org:act:40C4FBA5-455F-45AE-AD5A-33B6A6FB8723

*Sirindhorna khoratensis*, sp. nov.

urn:lsid:zoobank.org:act:54C342F2-EB92-4047-8F78-714025579CB5

### Etymology

Dedication to the Princess Maha Chakri Sirindhorn, Thailand, for her contribution to the support and encouragement of paleontology in Thailand. The specific name comes from the name of the locality, Khorat, which is the informal name of Nakhon Ratchasima Province, northeastern Thailand.

### Diagnosis

Basal hadrosauroid distinguished by an autapomorphy: sagittal crest extending along entire dorsal surface of the parietal and reaching the frontoparietal suture, and the following unique combination of characters: relatively straight frontoparietal suture, caudodorsally faced supraoccipital, no participation of the supraoccipital in the foramen magnum, antorbital fossa of the maxilla not visible, slightly rostrally deepening dentary ramus, simple troughs for dentary alveoli with vertical walls and tooth crown-shaped base, vertical coronoid process expanded along rostral and caudal margins, and dentary teeth with primary and secondary ridges but no accessory ridges.

### Holotype

An articulated braincase comprising the supraoccipital, exoccipitals, opisthotics, prootics, parietals, frontals, basioccipital, basisphenoid orbitosphenoids, parasphenoid and laterosphenoids, with postorbitals and squamosals (NRRU3001-166)

### Referred materials

Disarticulated elements of skull and mandibles: a braincase articulating with a left postorbital (NRRU-A2035), dorsal half of a braincase (NRRU3001-65), caudal portion of a braincase (NRRU3001-179), a right premaxilla (NRRU-A3623), a left maxilla (NRRU-A2048), a right maxilla (NRRU-A2047), a right jugal (NRRU3001-7), a right quadrate (NRRU3001-175), a predentary (NRRU3001-169), a left dentary (NRRU3001-14), a right dentary (NRRU3001-167), a right surangular (NRRU3001-137), isolated maxillary teeth (NRRU-A1956, A3630, A3649, NRRU3001-157, 163), an isolated dentary tooth (NRRU3001-28).

### Locality and horizon

In Ban (meaning “village”) Saphan Hin, Suranaree Subdistrict, Muaeng Nakhon Ratchasima District, Nakhon Ratchasima Province, Thailand. Lower Cretaceous (Aptian) Khok Kruat Formation.

## Description

**Fig 2:**
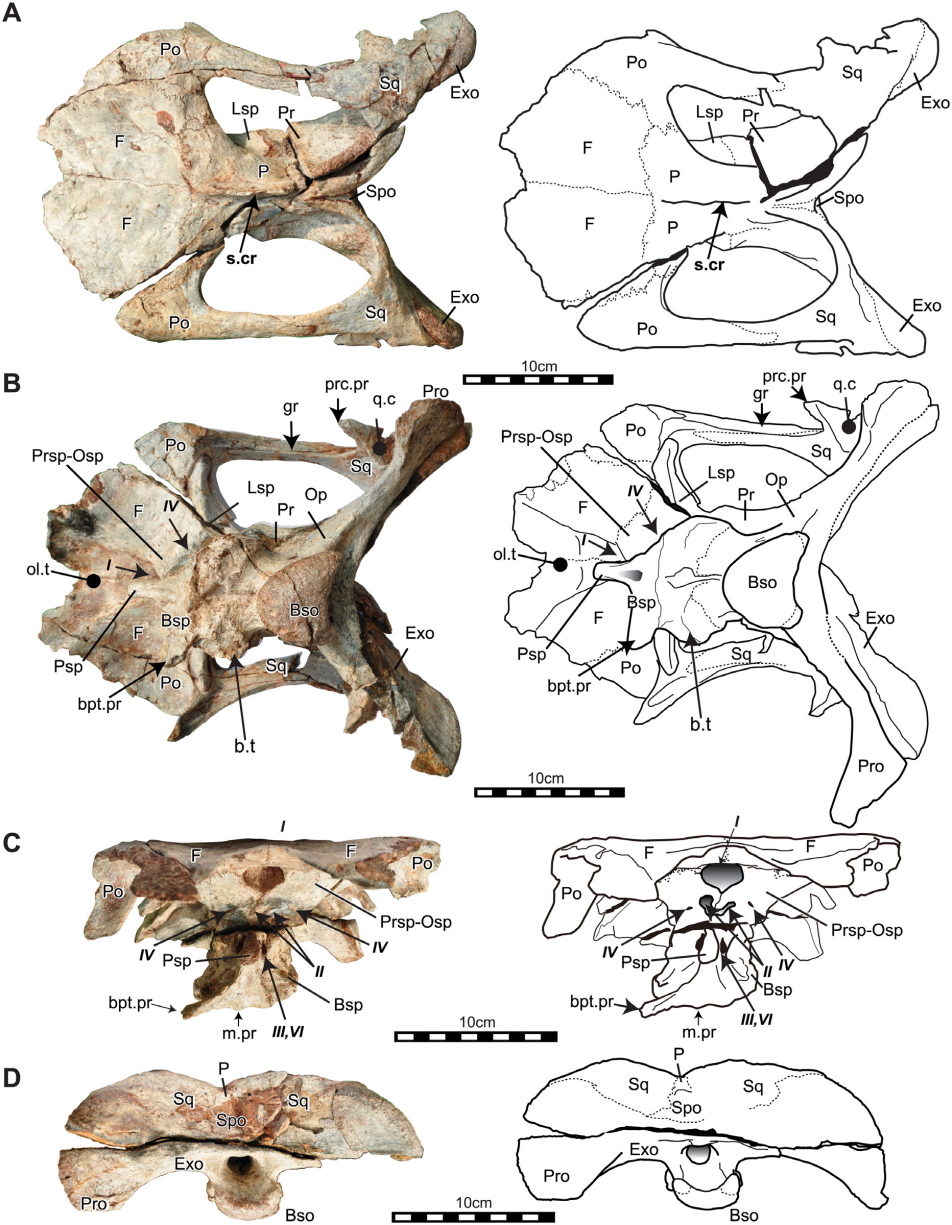
Articulated skull (braincase) of *Sirindhorna* (NRRU3001-166). In dorsal (A), ventral (B), rostarl (C), and caudal (D) views. Abbreviations: Bso, basioccipital; bpt.pr, basipterygoid process; Bsp, basisphenoid; b.t., basal tubera; Exo, exoccipital; F, frontal; i.c, groove for the inter carotid; Lsp, laterosphenoid; m.pr, median process; ol.t, olfactory tract; Op, opisthostic; Osp, orbitosphenoid; P, parietal; Pro, paroccipital; prcp.pr, precotyloid process; Po, postorbital; Pr, prootic; Prsp, presphenoid; Psp, parasphenoid; q.c, quadrate cotylus; Spo, spraoccipital; s.cr, sagittal crest; Sq, squamosal. Italic Roman number indicates cranial nerves. Left paroccipital process was lacked when taking photos. Scale bars equal 10 cm.

**Fig 3:**
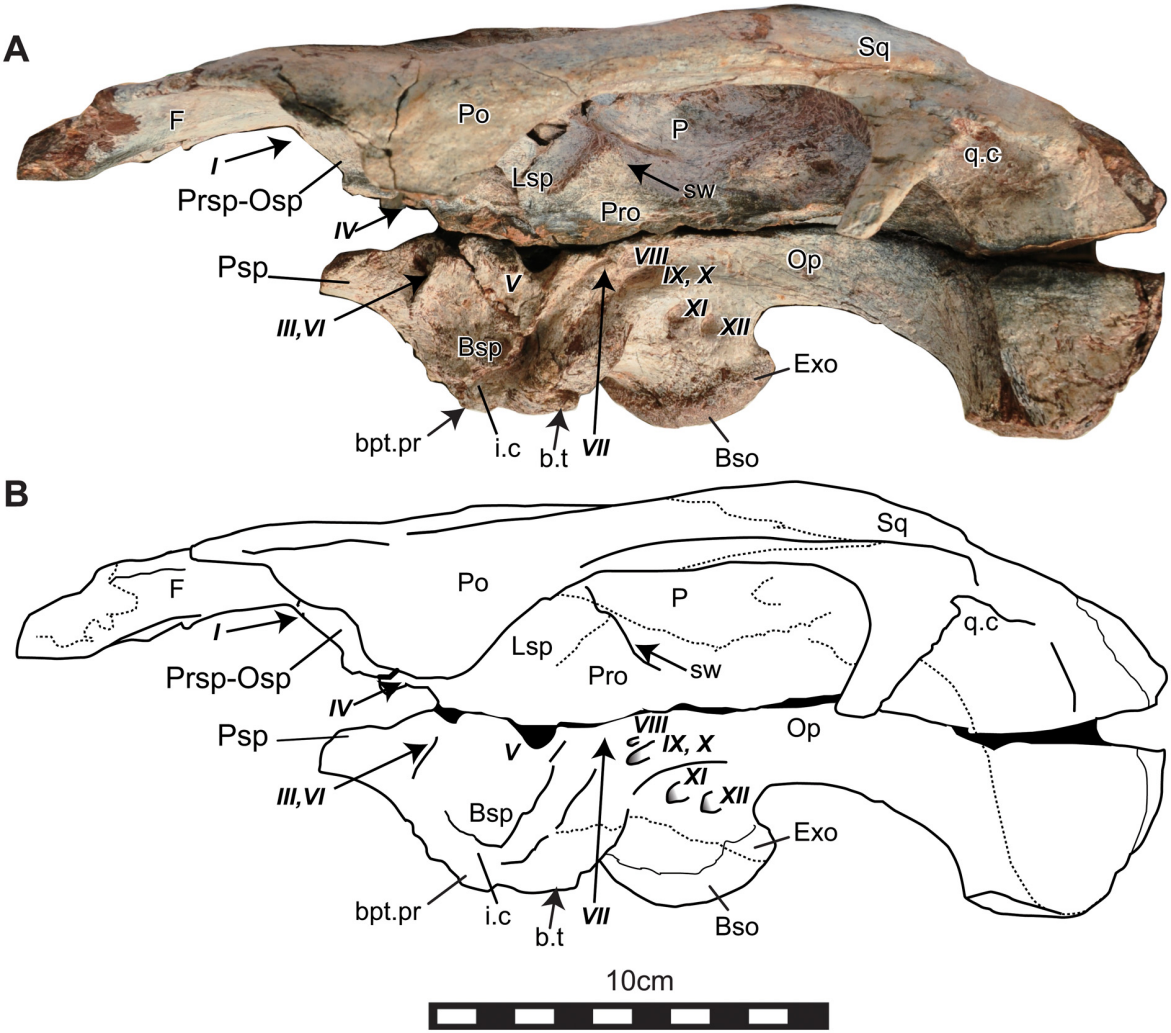
Photo (A) and line drawing (B) of the left lateral side of the skull (NRRU3001-166). Abbreviations: Bso, basioccipital; bpt.pr, basipterygoid process; Bsp, basisphenoid; b.t, basal tubera; Exo, exoccipital; F, frontal; i.c, groove for the inter carotid; Lsp, laterosphenoid; Op, opisthostic; Osp, orbitosphenoid; P, parietal; Pro, paroccipital; prcp.pr, precotyloid process; Po, postorbital; Pr, prootic; Prsp, presphenoid; Psp, parasphenoid; q.c, quadrate cotylus; s.cr, sagittal crest; Sq, squamosal; sw, swelling. Italic Roman number indicates cranial nerves. Scale bar equals 10 cm.

**Fig 4:**
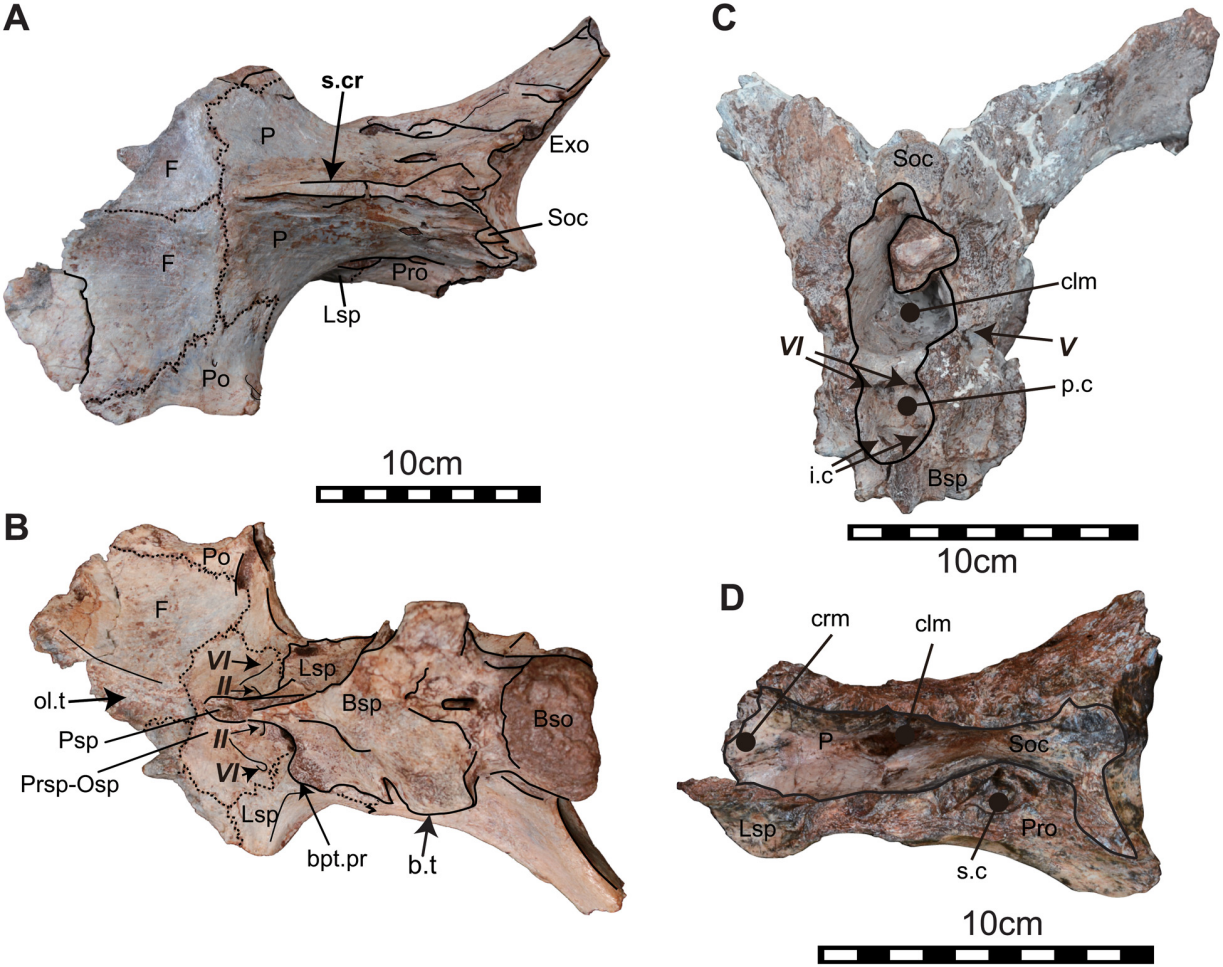
Other braincases. The articulated braincase (NRRU-A2035) in dorsal (A) and ventral views (B), the caudal portion of braincase (NRRU3001-179) in rostral view (C), and the dorsal part of braincase (NRRU3001-65) in ventral view (D). Abbreviations; Bso, basioccipital; bpt.pr, basipterygoid process; Bsp, basisphenoid; b.t, basal tubera; clm, cavity for the cerebellum; Exo, exoccipital; F, frontal; i.c, groove for the inter carotid; Lsp, laterosphenoid; Op, opisthostic; Osp, orbitosphenoid; P, parietal; p.c, pituitary cavity; Pro, paroccipital; prc.pr, precotyloid process; Po, postorbital; Pr, prootic; Prsph, presphenoid; q.c, quadrate cotylus; q.c, quadrate cotylus; s.cr, sagittal crest; Sq, squamosal. Italic Roman number indicates cranial nerves. Scale bars equal 10 cm.

**Fig 5:**
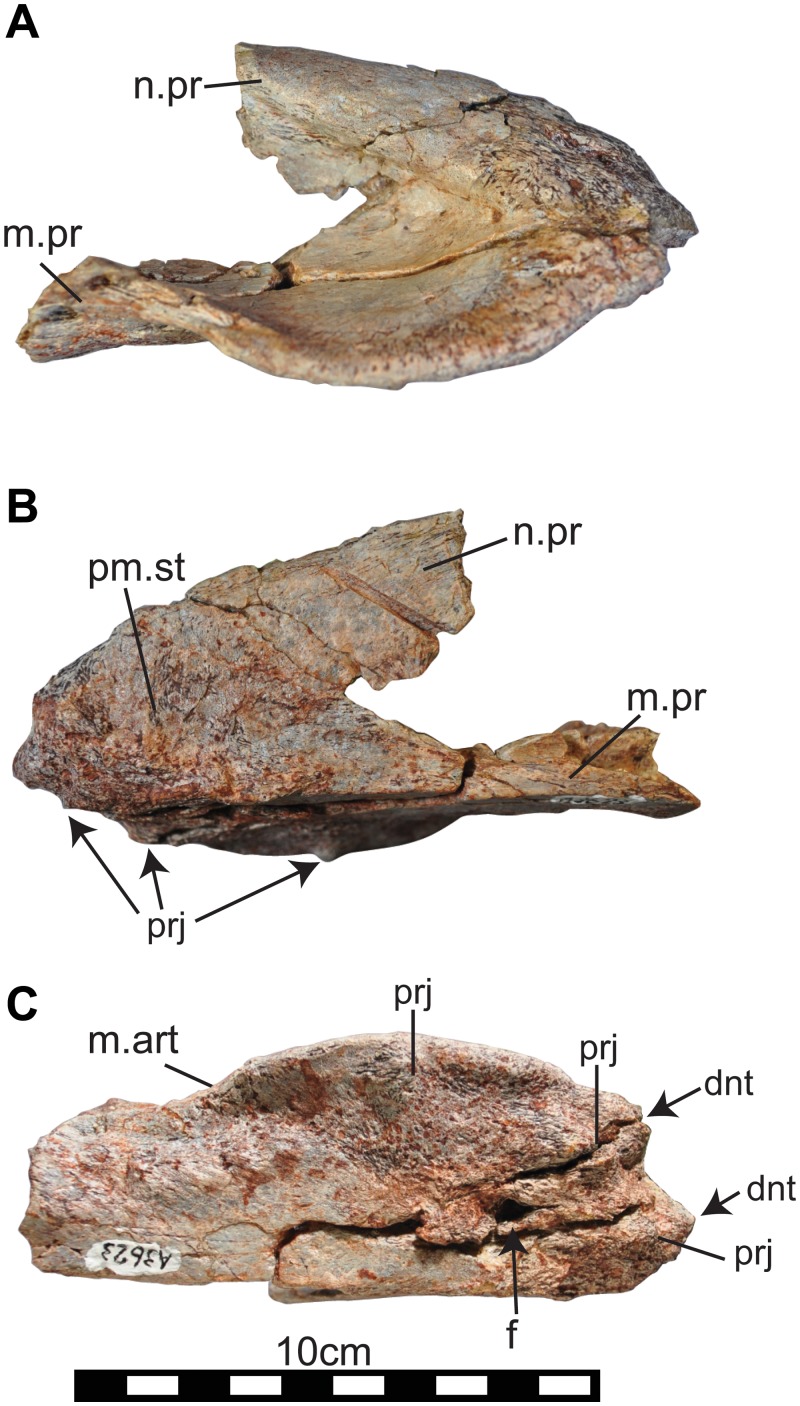
Right premaxilla (NRRU-A3623) of *Sirindhorna*. In lateral (A), medial (B), ventral (C) views. Abbreviations: dnt, denticulation; f, foramen; m.art, maxillary articulation; m.pr, maxillary process; n.pr, nasal process; pm.st, suture for the adjacent premaxilla; prj, projection. Scale bar equals 10 cm.

**Fig 6:**
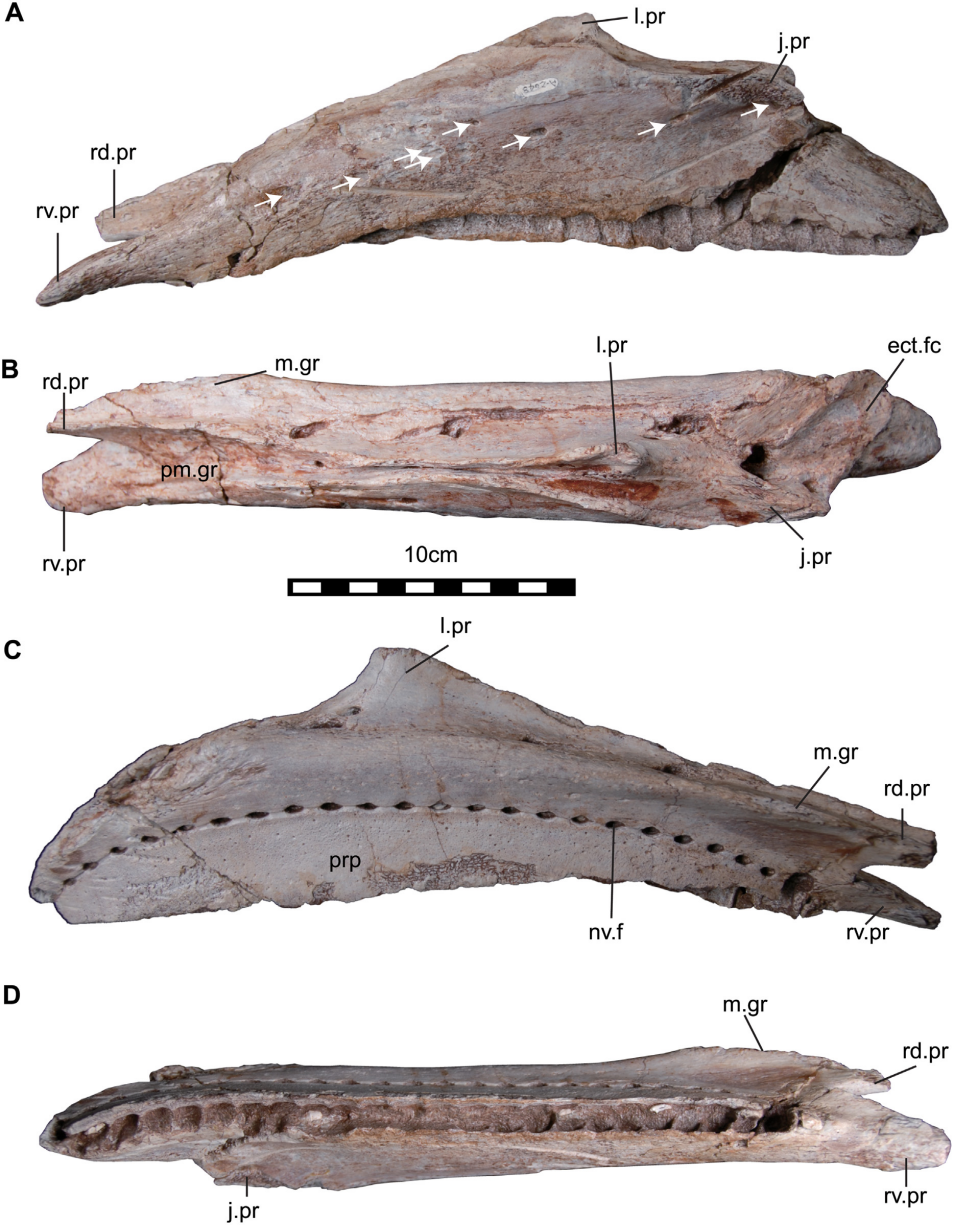
The left maxilla (NRRU-A2048) of *Sirindhorna*. In lateral (A), ventral (B), medial (C), occulusal (D) views. Abbreviations: ect.fc, ectpterigoid facet; j.pr, jugal process; l. pr, lacrymal process; m.gr, maxillary grooves; nv.f, neurovascular foramen; pm.gr, premaxillary groove; rd.pr, rostrodorsal process; rv.pr, rostroventral process. Scale bar equals 10 cm.

**Fig 7:**
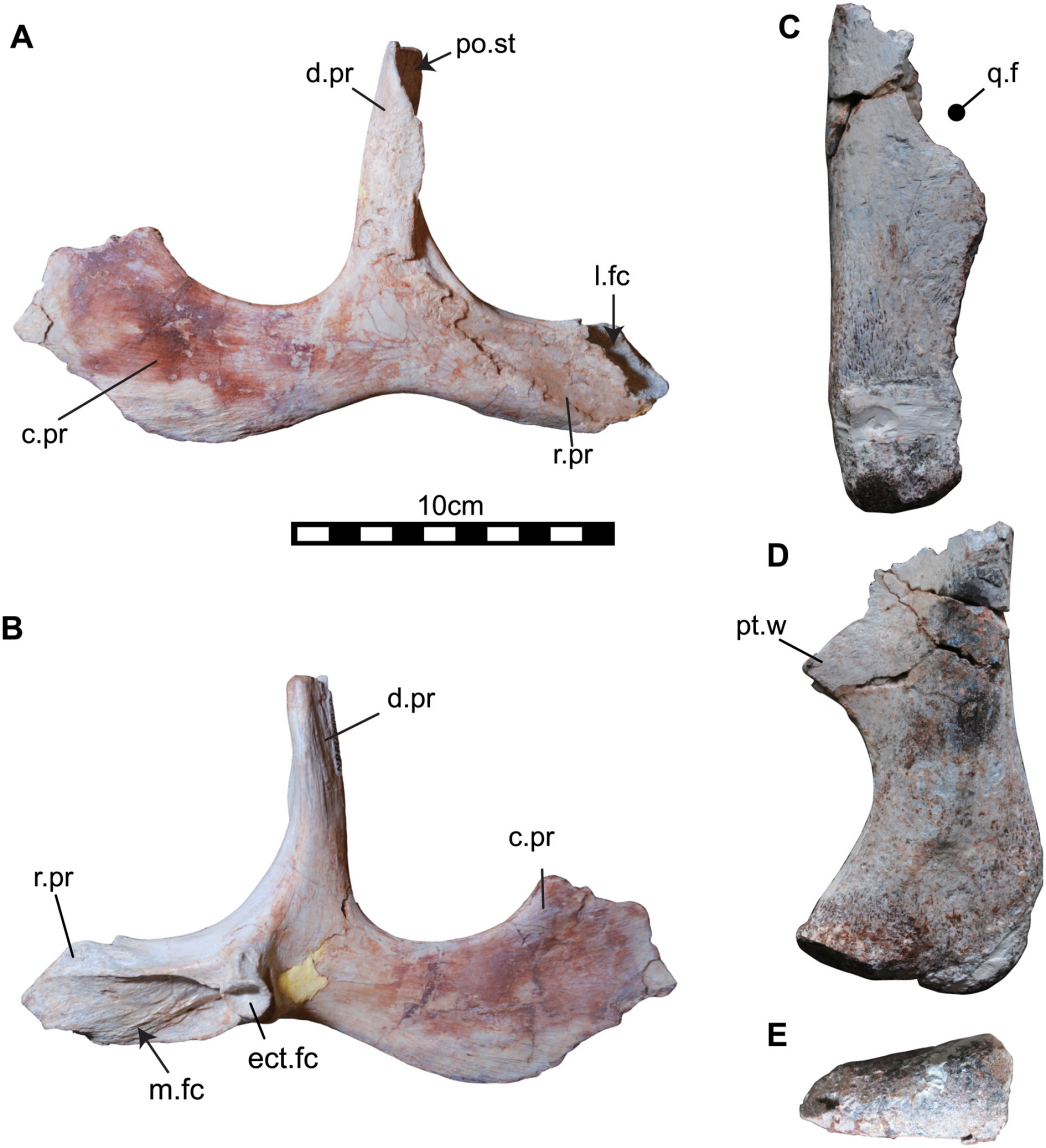
Right jugal (NRRU3001-7) and quadrate (NRRU3001-175) of *Sirindhorna*. The right jugal in lateral (A), medial (B) views. The right quadrate in lateral (C),caudal (D), and distal (E) views. Abbreviations: d.pr, dorsal process; ect.fc, ectpterigoidal facet; l.fc, lacrymal facet; m.fc, maxillary facet; po.st, postorbital suture; pt.w, pterygoid wing; q.f, quadrate foramen; r.pr, rostral process. Scale bar equals 10 cm.

**Fig 8:**
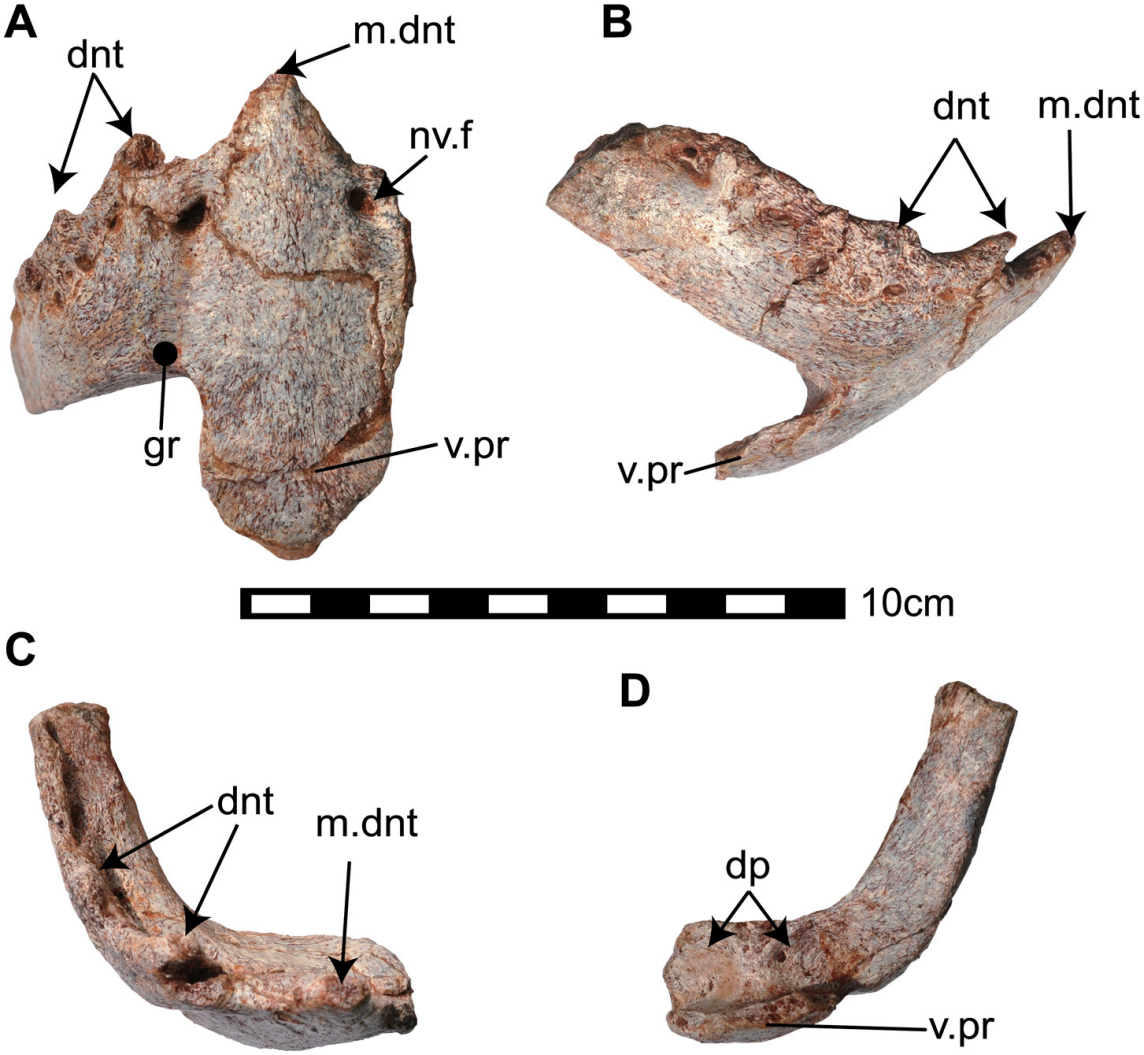
Predentary (NRRU3001-169) of *Sirindhorna*. In rostroventral (A), right lateral (B), occulsal (C), caudoventral (D) views. Abbreviations: dnt, denticle of the predentary; dp, depression; gr, groove; m.dent, median denticle; nv.f, neulovascular foramen; v.pr, ventral process. Scale bar equals 10 cm.

**Fig 9:**
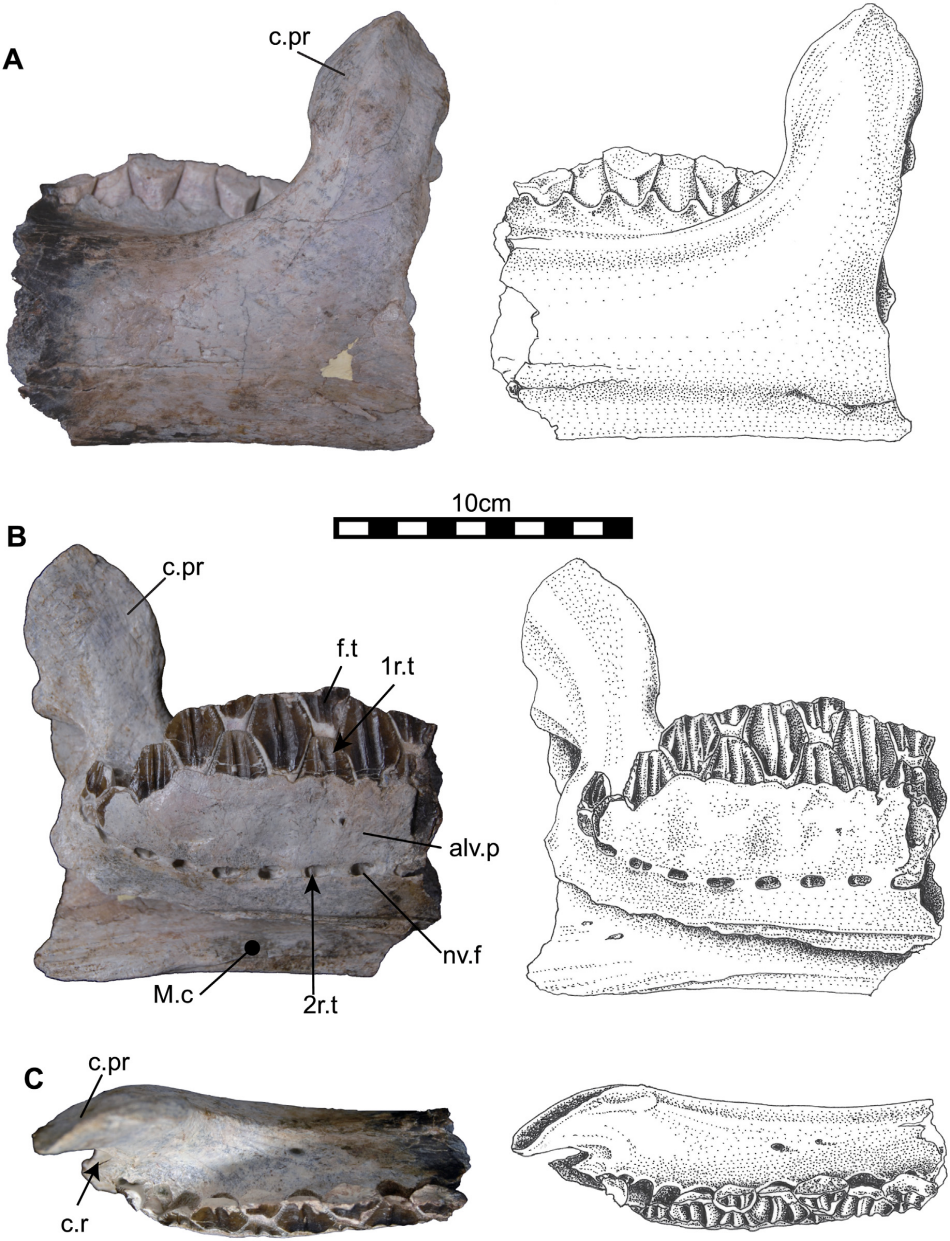
Caudal portion of left dentary (NRRU3001-14). In labial (A), occulusal (B), lingual (C) views. Abbreviations: alv.p, alveolar parapet; c.pr, coronoid process; c.r, cheek recess; M.c, Meckelian canal; nv.f, neurovascular foramen; 1r.t, first replacement tooth; 2r.t, second replacement tooth. Scale bar equals 10 cm.

**Fig 10:**
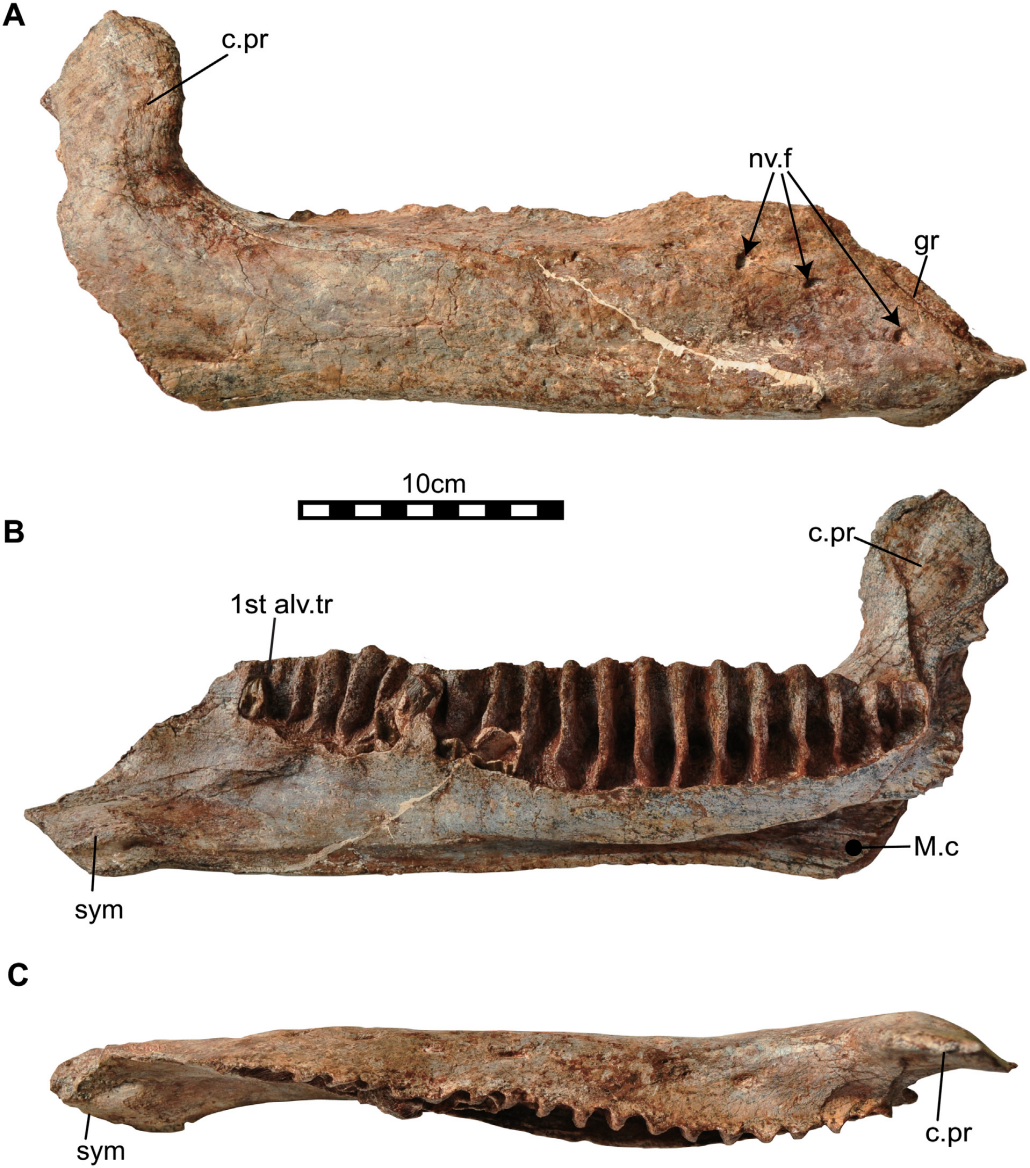
Right dentary (NRRU3001-167) of *Sirindhorna*. In labial (A), lingual (B), and occulusal (C) views. Abbreviations: alv.tr, alveolar trough; c.pr, coronoid process; gr, groove; nv.f, neulovascular foramen; sym, symphysis; v.pr, ventral process. Scale bar equals 10 cm.

**Fig 11:**
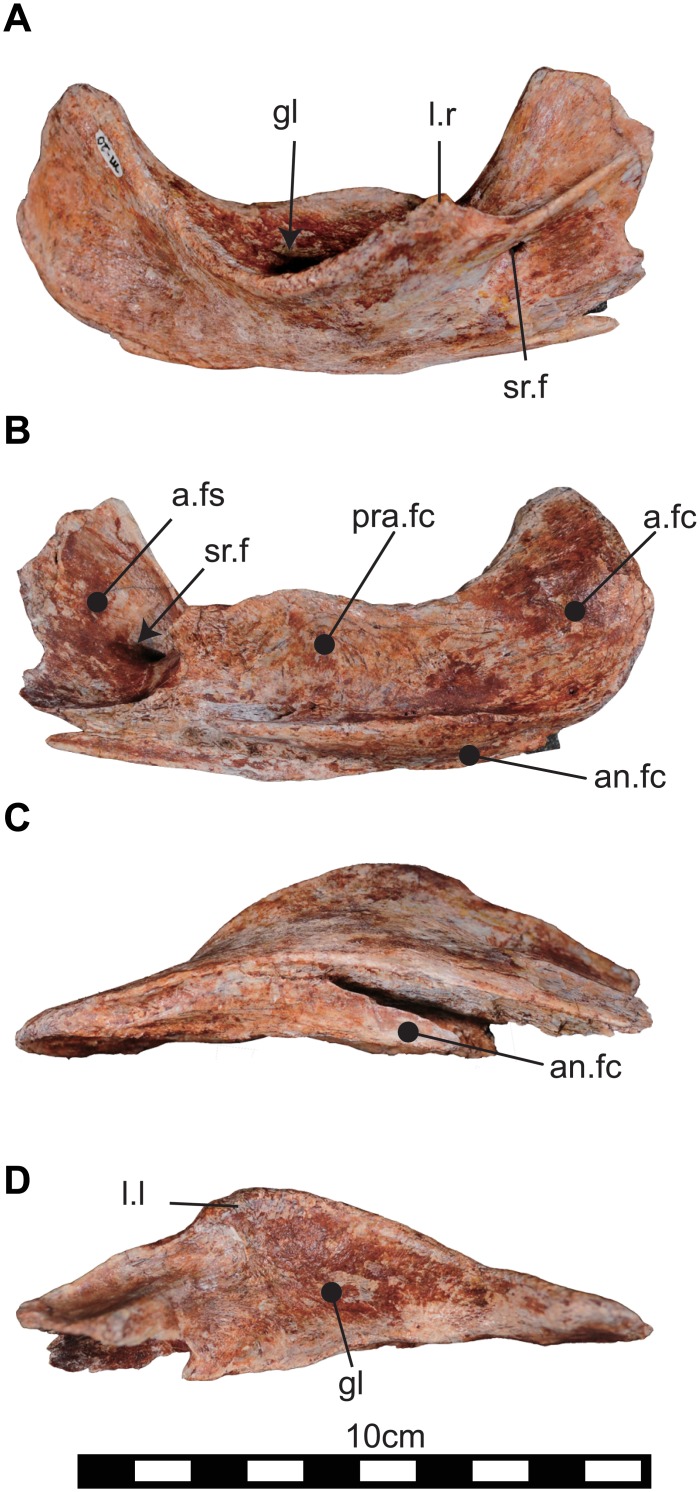
Right suranguar (NRRU3001-137) of *Sirindhorna*. In lateral (A), medial (B), ventral (C), and dorsal (D) views. Abbreviations: a.fs, adductor fossa; a.fc, articular facet; an.fc, angular facet; gl, glenod; l.l, lateral lip; sr.f, surangular foramen. Scale bar equals 10 cm.

**Fig 12:**
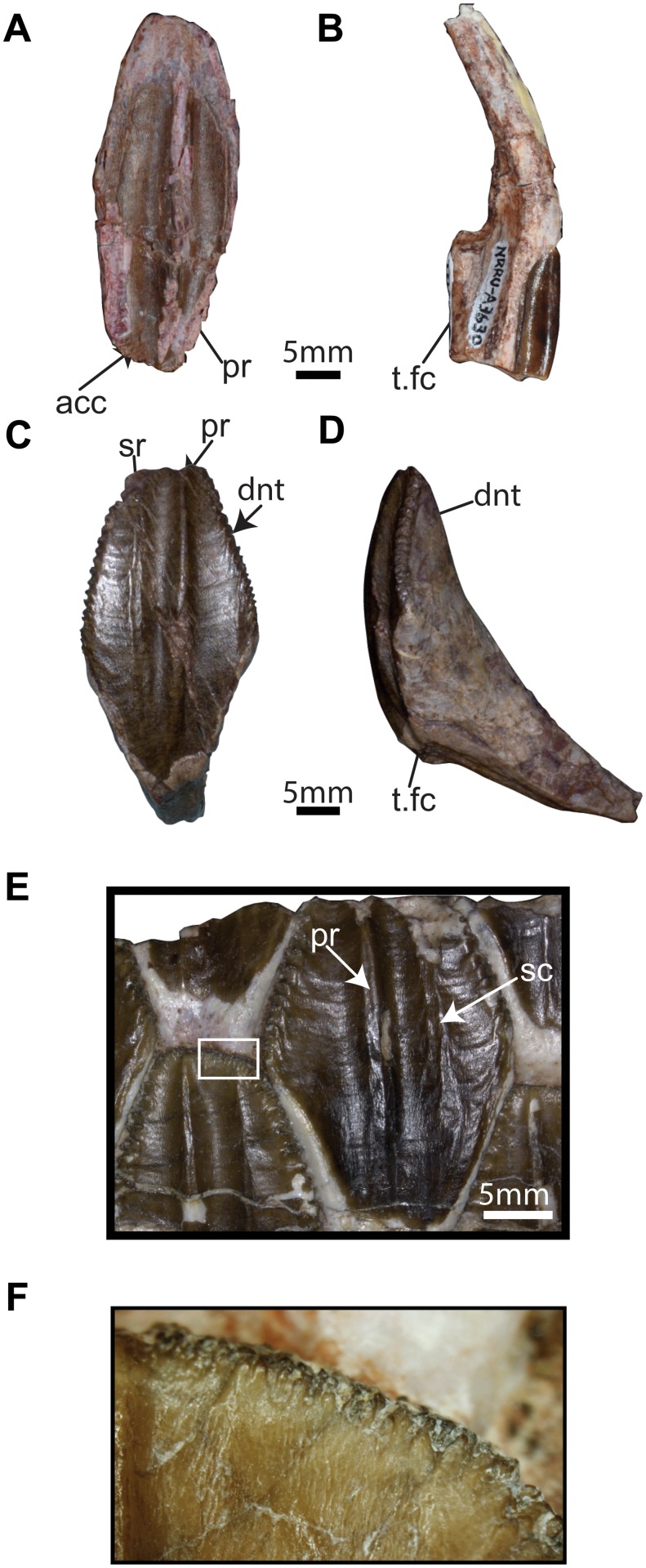
Maxillary and dentary teeth of *Sirindhorna*. Isolated left maxillary teeth in labial (A; NRRU-A1959) and mesial (B) views (NRRU-A3630). An isolated dentary tooth (NRRU3001-28) in lingual (C) and distal (D) views. Lingual side of 4—5th preserved teeth of the dentary (NRRU3001-14) (E) and mammilations on denticles (F) on the apical edge from white squared area in E. Abbreviations: acc, accesory ridges; dnt, denticle; ml, mammilation; pr, primary ridge; sc, secondary ridge; t.f, facet for an adjacent tooth. Scale bars equal 5 mm.

Elements of the cranial skeleton have been discovered in disarticulation and suffered from pre-and postmortern deformation. Braincases and dentaries have several duplications that indicate an inclusion of at least four individuals. Nevertheless, those duplicated elements do not show any diagnosable characters as distinct taxa. We, therefore, considered iguanodontians bones described here as one taxon. Three articulated cranial bones and the dorsal potion of a braincase were known. NRRU3001-166 is the best-preserved skull. This specimen was originally separated into dorsal and ventral portions along with a horizontal breakage line running through the foramen for the trigeminal nerves (V) when it was found. The cranial portions rostral to the frontals are disarticulated and missing (Figs 2 and 3). The ventral process of the left postorbital, the left basipterygoid process and the left paroccipital process are broken. The right paroccipital process was missed when photographing, but this part was found later and added in the line drawing (Fig 2B and 2D). NRRU-A2035 is an almost complete braincase preserved with an articulated piece of left postorbital. Half of the right frontal, the left opisthostic-exoccipital complex, both side of the paroccipital process, a part of the prarasphenoid, basisphenoid and basioccipital are missing (Fig 4A and 4B). Descriptions of braincase include characters in this specimen. NRRU3001-65 and 179 are the dorsal half and the caudal part of braincases, respectively (Fig 4C and 4D). Although these two braincases show dorsal and caudal portions of brain cavities, we will report the detail structure of the braincase morphology of this new taxon in a separate paper. Other cranial parts: dermatocranium, mandible and teeth, are also described here (Figs 5–12). All measurements of bones are seen in Table 1.

**Table 1:** Measurements of described materials from Ban Saphan Hin.

Element	Specimen Number	Side	Length (mm)	Width (mm)	Height (mm)
Skull roof	NRRU3001-166	-	292	198 / 260^a^	120
Skull roof	NRRU-A2035	-	232	124	14
Skull roof	NRRU3001-65	-	137	88.8	64.2
Skull roof	NRRU3001-179	-	126	135	130
Premaxilla	NRRU-A3623	R	125	48.6	51.7
Maxilla	NRRU-A2048	L	321	47.2	84.1
Maxilla	NRRU-A2047	R	235+	35.3	76.0+
Jugal	NRRU3001-7	R	206	35.2	111
Quadrate	NRRU3001-175	R	28.6	59.1	128
Predentary	NRRU3001-169	-	73.6	49.7	82.2
Dentary	NRRU3001-14	L	138	49.0	146
Dentary	NRRU3001-167	R	336	39.6	141
Surangular	NRRU3001-137	R	115	34.1	53.1
Maxillary tooth	NRRU-A3630	R	14.4	14.1	47.8 / 17.0^b^
Maxillary tooth	NRRU-A1959	L	16.2	17.1	41.4 / 35.3^b^
Maxillary tooth	NRRU-A3649	L	13.2	16.2	62.0 / 40.4^b^
Maxillary tooth	NRRU3001-163	R	13.9	15.8	51.7 / 21.6^b^
Maxillary tooth	NRRU3001-157	L	13.3	14.5	37.4 / 31.5^b^
Maxillary tooth	NRRU3001-75	R	11.5	13.3	32.9 / 18.2^b^
Maxillary tooth	NRRU3001-76	R	17.4	17.2	46.5 / 26.7^b^
Dentary tooth	NRRU3001-28	R	13.3	16.7	49.1 / 29.4^b^

^a^Width between the postorbitals / width between the paroccipitals

^b^Height of the total / hight of the crown

### Skull

#### Frontal

The intact frontals meet along the midline with a distinct suture line. In dorsal view, the frontal is rostrocaudally elongated and pentagonal in shape and is sutured to the postorbital caudolaterally and to the parietal caudally. The frontoparietal suture is transversely straight (Figs 2A and 4A). In ventral view (Figs 2B and 4B), the laterosphenoid and orbitosphenoid surround the caudomedial margin on the ventral surface of the frontal. The suture between the frontal and orbitosphenoid ends at the fenestra for the olfactory nerve (I) (Fig 2B and 2C). The trough on the ventral surface of the frontal and along the interfrontal suture becomes transversely broader rostrally, forming the olfactory tract (Fig 2B).

#### Parietal

The elongated parietal exhibits a strong median sagittal crest on the dorsal surface; the crest extending rostrally to the frontoparietal suture (Figs 2A and 4A). In dorsal view, the parietals meet the frontals rostrally, the postorbitals rostrolaterally, the laterosphenoids rostroventrally and the prootic-opisthostics caudoventrally. There is a gentle swelling at the sutural line between this bone and the laterosphenoid (Fig 3). This swelling extends to the foramen for the trigeminal nerve (V) and is the boundary between fibers of the *adductor mandibulae externus profundus* and *the psuedotemporalis* as in modern reptiles [32]. In caudal view (Fig 2D), both sides of the parietal form a sharp-angled roof above the supraoccipital. The base of this parietal roof is sutured against the supraoccipital.

#### Postorbital

The postorbitals are articulated with the skull roof, and the ventral process of the left postorbital is missing (Fig 2B). The postorbital is triradiate and meets the frontal rostromedially, the parietal caudomedially and the laterosphenoid ventrally. The slender caudal process for the squamosal, which is a subtriangular in section, extends past the rostrorlateral margin of the supratemporal fenestra (Fig 2A and 2C), and bears a shallow groove running rostrocaudally on the ventral surface (Figs 2B and 4B). The ventral process for the jugal is projected rostroventrally and the rostral margin of this process forms the caudal portion of the orbit (Figs 2B, 2C and 3). The rostroventral surface of the postorbital is rounded and deeply concaved at the base of the ventral process (Figs 2C and 3).

#### Squamosal

The squamosals are preserved and tightly fused to adjacent bones (Fig 2). Each squamosal is quadradiate and comprises the dorsocaudal margin of the infratemporal fenestra. The rostral process for the postrorbital is slender and interfingers with the caudal process of the postorbital (Figs 2A, 2B and 3). The groove on the ventral surface of the postorbital process continues on the rostrodorsal surface of the ventral process (precotylar process) of the squamosal, forming the dorsal border of the infratemporal fenestra (Figs 2B and 3;[32]). This groove is the origin of M. *adductor mandibulae externus superficialis*, as seen in *Iguanacolossus* [33]. Caudal to the precotylar process, there is a glenoid fossa for the dorsal end of the quadrate, called a quadrate cotylus (Figs 2B and3; [32]). The caudomedial process curves rostrally, and extends onto the parietal and to the caudal end of the sagittal crest. The rostral surface of the caudomedial process is concave deeply. The squamosals do not meet along the sagittal line, and a narrow band of the parietal is visible in dorsal and caudal views (Fig 2A and 2D). The squamosal sits on the paroccipital process dorsally and the prootic-opisthostic complex mediodorsally. In caudal view, the squamosal is sutured to the parietal and the supraoccipital medially (Fig 2D).

#### Presphenoid and orbitosphenoid

No suture between presphenoid and orbitosphenoid is preserved in discovered braincases. According to hadrosaurids’ braincases in [32], these bones meet rostrally on the ventral surface of the frontals, and contact the laterosphenoids caudally, and the basisphenoid ventrally (Figs 2B, 2C and 4B). The foramen for the optic nerve (II) might be situated on the caudolateral border of this complex and close to the suture with the laterosphenoid (Fig 2C).

#### Laterosphenoid

The laterosphenoid is situated between the orbitosphenoid and prootic, and forms the rostrolateral side of the braincase (Figs 2, 3 and 4). The robust rostrolateral process articulates with the medial side of the postorbital. The suture with the prootic extends ventrally to the rostral part of the foramen for the trigeminal nerve (V) (Figs 2B, 3 and 4).

#### Parasphenoid

The parasphenoid forms the rostroventral portion of the braincase (Figs 2, 4A and 4B). It meets the orbitosphenoid rostrodorsally, and its caudal part attaches to the basisphenoid dorsally. The cultriform and basiptergoid processes are not well preserved.

#### Prootic

The prootic meets the laterosphenoid rostrally, the parietal dorsally, the basisphenoid ventrally and the opisthotic caudally (Figs 2C, 4A and 4B). The foramen for the trigeminal nerve (V) penetrates at the rostroventral corner of the prootic. The caudoventral corner of the prootic seems to contribute to the rostral half of the foramen for the auditory nerve (VIII), although no distinct suture is visible. The prootic and the opisthotic are completely fused to each other and to the exoccipital.

#### Basisphenoid

The basisphenoid is deformed and broken (Fig 2B and 2C). It meets the orbitsphenoid rostrodorsally, the laterosphenoid dorsally, the prootic caudodorsally and the basioccipital caudally. No distinct suture with the laterosphenoid is observable. The left basipterygoid process is missing. A deep groove extends ventrally from between the foramina for the trigeminal (V) and facial (VII) nerves, and it turns rostrally dorsal to the basal tubera (Fig 3). This groove is possibly for the internal carotid artery and palatine branch of the facial nerve [34]. NRRU3001-179 shows part of internal structure of the braincase (Fig 4C). There are two foramina on the dorsal side of the pituitary cavity, which are possibly for the abducens nerve (VI). The other two foramina on the ventral side, which are probably the openings of internal carotid artery [32].

#### Supraoccipital

The supraoccipital is trapezoidal and sutured to the parietals dorsally, the squamosals laterally, and the exoccipitals ventrally (Figs 2D and 4A). The ventral edge of the supraoccipital does not extend to the foramen magnum, which is seperated by the exoccipitals. The dorsal surface of the supraoccipital faces caudally.

#### Exoccipital

The exoccipitals forms the entire margin of the foramen magnum, except for a ventral narrow groove where the basioccipital is exposed (Fig 2D). Dorsal to the foramen magnum, the distinct exoccipital shelf bears a small bump on its ventral surface. The caudoventral swellings of the exoccipitals form the occipital condyle together with the basioccipital. The paroccipital process is directed ventrally. There are foramina for the glossopharyngeal (IX), vagus (X), accessory (XI) and hypoglossal nerves (XII) arranged almost horizontally in the opithostic-exoccipital complex (Fig 3).

#### Basioccipital

The basioccipital is slightly deformed but well preserved. It meets the exoccipitals dorsolaterally and the basisphenoid rostrally. The heart-shaped occipital condyle consists of the basioccipital at the ventral and exoccipitals at the dorsaolateral sides and is caudroventrally oriented (Figs 2B, 3 and 4B).

#### Premaxilla

The right premaxilla (NRRU-A3623) is missing large portions of its nasal and maxillary processes (Fig 5). In lateral view, the laterally expanded oral margin extends slightly below the level of the maxillary process. The oral margin and the rostral part of the nasal process show a rugose surface, indicating the attachment of the keratinous sheath. The narial fossa between the nasal and maxillary processes caudal to the main body of the premaxilla is broken (Fig 5A). In medial view, the symphysial surface at the main body of the premaxilla presents sutural grooves running rostroventrally from the base of the nasal process (Fig 5B). The medial surface of the nasal and maxillary processes is flat. In ventral view, the rostral margin of the premaxilla bears two distinct denticles (Fig 5C). The ventral surface of the body of the premaxilla bears three blunt projections and one large foramen (Fig 5B and 5C). There is a shallowly concave articular surface with the rostroventral process of the maxilla on the ventral surface of the maxillary process caudal to the oral margin.

#### Maxilla

The left maxilla (NRRU-A2048) is completely preserved (Fig 6). The rostral part of the maxilla is bifurcated and forms the rostrodorsal and rostroventral processes. The apex of the sub-triangular lacrimal (or ascending) process is located at the two fifths of the total length from the caudal end, and the rostrocaudally elongated maxillary body relative to the height of that apex make the maxilla a low-angled triangle-shape in lateral view (Fig 6A). The lateral surface of the maxilla bears several foramina and the rostral-most one is the largest. The ventral marginal line of the maxilla is almost straight and terminates in the rostroventral process. The bifurcated rostral part of maxilla forms a groove for the articulation of the maxillary process of the premaxilla, which becomes narrower toward the lacrimal process. There is no evidence of the antorbital fossa (fenestra), and the articular surface for the jugal is situated just caudal to the lacrimal process. The jugal process is missing its lateral projection, and probably does not form the long and robust caudolateral projection, which fits into a hollow formed in the medioventral surface of the rostral process of the jugal. The caudal end of the jugal process bears a deep groove running dorsoventrally that houses a foramen penetrating rostroventrally (Fig 6A). In dorsal view, the body of the maxilla is nearly straight, and the lateral and medial outlines of the maxilla run parallel (Fig 6B). There is the large ‘tongue’-shaped ectopterygoid facet caudal to the jugal process. In medial view, the medial surface of the maxilla bears an arched row of 24 subcircular neurovascular foramina, called “special foramina” (Fig 4C; [35]). A shallow groove connecting these adjacent foramina is inferred to be the accommodation of the dental lamina [35]. Caudomedial to the medial side of the rostrodorsal process, the maxillary grooves are weakly developed (Fig 6C). In ventral view, twenty-four alveoli are rostrocaudally arranged and slightly curved caudolaterally, and five of them preserve functional teeth (Fig 6D).

#### Jugal

The right jugal (NRRU3001-7) is almost complete, lacking only the dorsal tip of the caudal process (Fig 7A and 7B). The triradiate jugal consists of three processes: a rostral process that contacts the maxilla and the lacrimal, a dorsal process that articulates with the postorbital, and a caudal process that meets the quadratojugal. The ventral border of the jugal is weakly bowed. The rostral process of the jugal is slightly curved rostrodorsally and does not deepen dorsoventrally. The rostral process terminates in the articular facets for the lacrimal on the lateral aspect and for the maxilla on the medial side. The dorsal process of the jugal extends almost perpendicular to the long axis of the jugal and ends at the articular facet for the postorbital, becoming broader mediolaterally. A lateral skirt of the dorsal process covers the jugal process of the postorbital in articulaton. In medial view, the rostral process bears the medially facing articular facet for the maxilla, which is heavily striated and separated into two fossae at its caudal end (Fig 7B). This facet deepens caudally and extends into the interior of the jugal, lateral to the facet for the ectopterygoid. The facet for the ectopterygoid is deep and oval-shaped, and positioned just rostral to the base of the dorsal process (Fig 7B). This facet is bounded just caudal to the facet for the maxilla by a thin ridge. The mediolaterally thin caudal process becomes dorsoventrally deep caudally and bears a shallow and broad facet for the quadratojugal.

#### Quadrate

The right quadrate (NRRU3001-175) is missing its dorsal half (Fig 7C–7E). The body of the quadrate is dorsoventrally straight in lateral view, and the jugal wing (the rostral projection) is weakly developed. The ventral border of the paraquadrate foramen is preserved. The caudal surface of this bone is relatively flat and expanded at the ventral end. A strongly curved medial border continues to the pterygoid wing in caudal view (Fig 7D). The ventral condyle is subtriangular shaped in ventral view (Fig 7E).

### Mandible

#### Predentary

A single predentary (NRRU3001-169) is preserved, missing its left caudal process, the caudal end of the right caudal process, and most of the ventral process (Fig 8). The predentary is robust and relatively deep, forming a horseshoe-shape in occlusal view. The caudal processes are divergent caudolaterally. Dorsally directed spike-like denticles are present on the rostrodorsal margin; the median denticle is the largest and most strongly pointed (Fig 8A). The other denticles decrease in size caudally to the second and third one (Fig 8A and 8B). Ventral to the row of denticles are neurovascular foramina and grooves probably associated with the keratinous sheath (Fig 8A and 8B). In rostral view, the rostral foramina are the largest and penetrated at the base of the median denticle. The deep groove on either side runs into the rostral foramina. The ventromedial process projects caudoventrally, and the bifurcated projections on the ventral tip is not preserved (Fig 8A and 8B). The ventral side of the right caudal process of the predentary is relatively flat, although there is a shallow depression on each side close to the base of the ventral process where the rostral tip of the dentary fits (Fig 8D).

#### Dentary

Two dentaries have been unearthed from the site, one is a left dentary with well-preserved teeth in situ but the rostral part of the dentary is missing (Fig 9; NRRU3001-14); the other is a nearly complete right dentary which lacks observable teeth (Fig 10; NRRU3001-167). The following description is based on these two specimens. The dentary consists of a deep dentary ramus and a thumb-shape large coronoid process in lateral view (Figs 9A and 10A). The ventral edge of the dentary ramus is nearly straight and the ventral surface of the symphyseal region is visible (Fig 10A). The coronoid process is relatively robust and large. It is slightly expanded along its rostral and caudal margins with a pointed dorsal tip. The coronoid process is subvertical on both specimens (Figs 9A and 10A). The lateral surface of the dentary is nearly flat at the rostral part but shows convexity at the base of the coronoid process. In medial view (Fig 10B), a short diastema exists between the first alveolar trough and the predentary articulation [36]. The symphysis faces rostromedially at the rostral tip of the dentary. The thin alveolar parapet covers over more than half of the dorsoventral depth of each tooth family (Fig 9B). There are seven completely preserved and two broken neurovascular foramina recognizable on the medial side (Fig 9B). The foramina are linked by weak grooves, which probably marks the passage for the main blood vessel and sensory nerve trunk [35]. One functional and two replacement teeth are housed in each tooth family. However, the 2^nd^ replacement tooth crown is rudiment with undeveloped enamel and can be seen through the neurovascular foramen (Fig 9B). There are twenty alveoli and five incomplete replacement teeth preserved in the right dentary (Fig 10B). The alveoli are simple grooves, but are shaped like the crown of a tooth at the base (Fig 10B). The dorsoventral depths of the alveoli decrease rostrally and caudally, and are greatest at the middle of the tooth row around 12^th^ alveolus. The caudal end of the tooth row is positioned rostral to the apex of the coronoid process on both specimens. Ventral to the alveolar parapet, there is a deep and large groove called the Meckelian canal (Figs 7B and 8B). In dorsal view, the tooth row slightly curves medially at the base of the coronoid process (Figs 9C and 10C). Although the right dentary preserves no functional teeth, the row of alveoli, which corresponds to the tooth row, is also slightly arched medially (Fig 10C). The cheek recess (or buccal shelf) between the coronoid process and the caudal-most alveolar trough is present (Figs 9B and 10C).

#### Surangular

A single right surangular (Fig 11; NRRU3001-137) is known, although the rostral portion articulated is missing. There is a small surangular foramen positioned rostroventral to the glenoid on the lateral surface of this bone (Fig 11A). The lateral lip extends rostrodorsally and forms a narrow horizontal shelf. The portion caudal to the glenoid becomes hooked dorsally and compressed transversely (Fig 11A and 11D); the articular would have contacted the medial side of this part (Fig 11B). The angular was possibly visible in lateral view when it was articulated with the surangular (Fig 11A). Medially, the adductor fossa is developed rostral to the glenoid, and the surangular foramen is visible (Fig 11B). The ventral surface of the surangular (Fig 11C), where the angular fits, is relatively flat and rostrally bifurcated.

### Dentition

#### Maxillary tooth

Five isolated maxillary teeth have been collected and two of them are shown for description (NRRU-A3630 and A1959). Basically, the crown of tooth is apicobasally elongated lanceolate shape and enameled on the labial side (Fig 12A). The prominent primary ridge separates the labial surface unevenly. The distal portion of the labial surface bears weak subsidiary ridges and slightly broader than the mesial portion. The mesial and distal margins are denticulated at the apical half. An unenamelled lingual side of the crown is more or less thick and sub-angled (Fig 12B). The lingual side becomes mesiodistally compressed in basal direction and bears a depression on both mesial and distal sides. The root of the maxillary tooth is relatively slender and arched lingually. The labial side of the root is rounded, whereas the lingual side is grooved for the replacement tooth.

#### Dentary tooth

Dentary teeth are well preserved and tightly fitted into each other to form a dental battery in the left dentary (Fig 9). The occlusal plane is flat and faces labiodorsally (Fig 9A). The caudal seventeen teeth are observable in lingual view, though no teeth are completely exposed because the alveolar parapet covers the lower half of the dentitions (Fig 9B). The caudal-most tooth is marked by its extremely small size and abnormal shape relative to the other teeth. To describe dentary teeth, one isolated tooth (NNRU3001-28) is employed here (Fig 12C and 12D). The crown of the tooth is moderately wide leaf-shaped (the ratio of apicobasal length / mesiodistal width: 1.9). The distal offset of the primary ridge makes the crown asymmetric (Fig 12C and 12E). The secondary ridge is positioned mesial to and is less prominent than the primary ridge. There are no other accessory ridges on the crown. Denticles are present on the mesial and distal margins of the upper half of the crow and become smaller towards the apex of the crown. Each denticle bears tiny mammilations (Fig 12E and 12F). Enamel covers only the lingual surface and marginal denticles of the crown.

## Comparisons

The osteological features, especially those in the oral and cheek regions, such as the transversely wide and edentulous premaxilla, divergent vascular grooves on the rostral surface of the predentary, and wear facets on the dentary consisting of one functional tooth crown, suggest *Sirindhorna* to be a typical non-hadrosaurid styracosternans [37]. The following comparisons were achieved based on observations from original materials: *Dakotadon lakotaensis* (SDSM 8656), *Proa valdearinnoensis* (AR-1-2012), *Fukuisaurus tetoriensis* (FPDM-V40), *Koshisaurus katsuyama* (FPDM-V9079), *Ratchasimasaurus suranareae* (NRRU-A2064), *Jinzhousaurus yangi* (IVPP V 12691), *Equijubus normani* (IVPP V 12534), and the dentary of *Probactrosaurus gobiensis* (IVPP V 20171); replicas: *Xuwulong yueluni* [11], and *Jintasaurus meniscus* [8], literatures: *Mantellisaurus atherfieldensis* [38], *Iguanodon bernissartensis* [34], *Altirhinus kurzanovi* [39], *Probactrosaurus gobiensis* [5], *Gongpoquansaurus mazongshanensis* [40,41], *Bolong yixianensis* [42], and *Siamodon nimingami* [16]. Although several basal styracosternans with well-preserved cranial material from North America, such as *Hippodraco scutodens* [33] and *Theiophytalia kerri* [43] are known, their morphological traits, including the antorbital fenestra of the maxilla, the largely exposed angular in lateral view and so forth, are totally different and distinguishable from those of *Sirindhorna*. Therefore, we do not include these taxa to mention in the following comparison section.

### Skull roof

**Fig 13:**
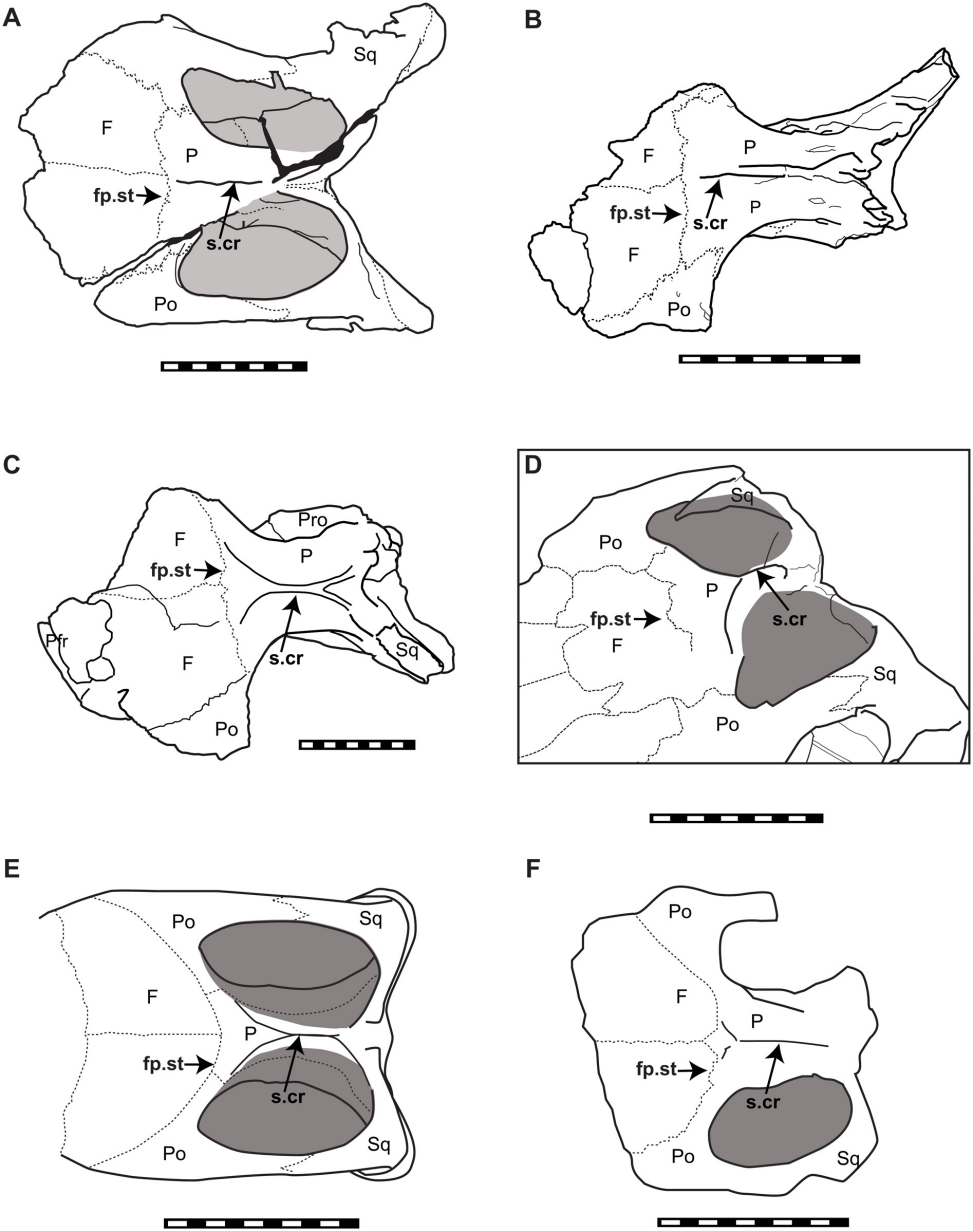
Comparison of the skull roof in dorsal view. (A) Holotype of *Sirindhorna* (NRRU3001-166), (B) referred material of *Sirindhorna* (NRRU-A2035), (C) *Dakodadon* (SDSM 8656), (D) *Jinzhousaurus* (IVPP-V12691), (E) *Mantellisaurus* (after [38]) and (F) *Probactrosaurus* (after [5]). Abbreviations; F, frontal; fp.st, frontoparietal suture; P. parietal; Po, Postorbital; Pro, prootic; s.cr, sagittal crest; Sq, squamousal. Scale bar equals 10 cm.

The configuration of the supratemporal fenestra is varied in iguanodontians (Fig 13). The rostrocaudally elongated oval-shaped supratemporal fenestra of *Sirindhorna* (Fig 13A and 13B) is similar to that in *Mantellisaurus* [38], *Proa* [44], *Probactrosaurus* [5] and *Levnesovia* [12], but different from the rostrolaterally directed oval-shaped supratemporal fenestra in *Lurdusaurus arenatus* [45], *Ouranosaurus nigeriensis* [46], *Equijubus* [47], *Jinzhousaurus* [48], *Xuwulong* [11], *Jintasaurus* [8] and possibly in *Yunganglong* [1], and the transversely wide one in *Gongpoquansaurus* [40]. However, the transversely straight frontoparietal sutural line of *Sirindhorna* is exceptional among those non-hadrosaurid styracosternans that having a “V”-shaped or rostrally excavated frontoparietal sutural line at the center (Fig 13). In addition, the sagittal (parietal) crest of the parietals extends along entire dorsal surface of the parietal, reaching the frontoparietal suture in *Sirindhorna*, but not in other non-hadrosaurid styacosternans (Fig 13).

### Upper jaw

The general morphology of the premaxilla with a transversely wide and a weakly downturned oral margin of *Sirindhorna* closely resembles to that in *Iguanodon*, *Mantellisaurus*, *Bolong*, *Equijubus*, and possibly *Jinzhousaurus* and *Xuwulong*. In contrast, it differs from those premaxillae with a robust structure and non-flared caudal portion of the oral margin in *Proa*, a deeply downturned lateral oral margin such as in *Fukuisaurus* and *Probactrosaurus*, and the transversely expanded maxilla in *Ouranosaurus* and other derived forms. The low triangular-shaped maxilla in lateral view of *Sirindhorna* indicates a resemblance to those of *Iguanodon* and *Mantellisaurus*, but *Sirindhorna* shows more elongated profile. This low-angled triangular shape and the caudally positioned lacrimal process of *Sirindhorna* is distinctive to the isosceles triangular shaped maxilla with the dorsally process positioned at the middle of the maxilla of *Siamodon* [16]. No visible antorbital fossa (or fenestra) of the maxilla in lateral view of *Sirindhorna* is a shared character with hadrosauriforms, but not with basal styracosternans. The shallow maxillary grooves are similar to that of *Koshisaurus* [15], but different from that of *Fukuisaurus* [14]. The jugals of *Sirindhorna* and *Mantellisaurus* [34] are similar in lacking the dorsoventrally expanded rostral processes and non-angular ventral border of their caudal processes. In contrast, the angular ventral border of the caudal process in *Jinzhousaurus* and *Xuwulong*, the rostrocaudally expanded maxillary facet on the medial side of the rostral process in *Altirhinus*, and the dorsoventrally expanded rostral process in *Ouranosaurus* are distinguishable from the equivalents in *Sirindhorna*. The quadrate is poorly preserved in *Sirindhorna*, but the weakly developed ventral border of the paraquadrate foramen is similar to that in *Altirhinus* and *Probactrosaurus*.

### Lower jaw

The predentary of *Sirindhorna* shows a ‘horseshoe’ shape with a slightly rounded rostral margin. The caudal process of the predentary is divergent almost caudally in dorsal view (Fig 8C). The divergent caudal processes of the predentary are seen in relatively primitive forms, such as *Camptosaurus*, *Proa*, and *Fukuisaurus*. The round rostral margin of the predentary bears one large median denticle and two small prominent denticles on each side in *Sirindhorna*, which resembles to those in *Altirhinus* and *Probactrosaurus*. In contrast, in *Proa*, *Mantellisaurus*, *Ouranosaurus*, *Bolong*, *Jinzhousaurus*, *Equijubus*, *Xuwulong*, *Eolambia* and more derived forms, the predentary has a subquadrate-shaped rostral margin in dorsal view or possesses no distinct large medial denticle. The dentary has a robust and straight dentary ramus with the subvertical coronoid process in *Sirindhorna* (Figs 9 and 10), which is similar to that in *Iguanodon*, *Mantellisaurus*, *Jinzhousaurus*, *Xuwulong*, *Equijubus* and *Probactrosaurus*. *Sirindhorna* has more robust and larger coronoid process relative to the dentary ramus as in *Equijubus*, *Eolambia*, and derived hadrosauriforms. The Thailand iguanodontian, *Ratchasimasaurus* and Chinese *Penelopognathus* have the relatively dorsoventrally short dentary ramus and caudally inclined coronoid processes, which are different from the condition in *Sirindhorna*. In *Sirindhorna* and other non-hadrosaurid iguanodontians, the surangular bears the surangular foramen.

### Dentitions

The maxillary tooth of *Sirindhorna* is similar to that of *Iguanodon*, *Mantellisaurus*, *Siamodon*, *Altirhinus*, and *Probactrosaurus*, bearing the lanceolate-shaped crown with a distinct primary ridge and weak subsidiary ridge on the labial surface. The crown of the maxillary tooth bears several or strong subsidiary ridges in *Lanzhousaurus*, *Bolong*, *Equijubus* and *Jinzhousaurus*, and has no subsidiary ridge in derived forms such as hadrosaurids. One tooth in each tooth family was functional in *Sirindhorna* as in basalmost iguanodontians, while *Altirhinus*, *Probactrosaurus* and other hadrosauriforms have at least two functional teeth. The dentary tooth of *Sirindhorna* is mesiodistally wider than the maxillary tooth and shows a leaf-shaped crown covered by thin enamel with one primary and several weak subsidiary ridges, as seen in *Iguanodon*, *Mantellisaurus*, *Fukuisaurus*, *Ouranosaurus*, and *Altirhinus*. *Equijubus* bears no distinct primary but several subsidiary ridges, *Probactrosaurus* possesses lanceolate shape crown, and *Gongpoquansaurus* does not have subsidiary ridges. In *Sirindhorna*, one functional and two replacement teeth are housed in each alveolus of the dentary. The 2^nd^ functional tooth is still rudiment as in *Altirhinus* and *Equijubus*, which seems to be a transitional condition.

The above comparisons indicate *Sirindhorna* is probably more derived than *Iguanodon* and *Mantellisaurus*, and close to Asian basalmost hadrosauroids.

## Phylogenetic Analysis and Discussion

To recover the phylogenetic position of *Sirindhorna*, we code *Sirindhorna* into the data matrix of [37], which employed well-preserved 27 taxa and 105 characters. The data of *Sirindhorna* is shown in S1 File. The data matrix were reassembled using Mesquite v.3.01 [49] and analyzed in TNT [50]. The analysis was performed using the traditional search with the tree bisection reconnection algorithm: all characters were treated as unordered and unweighted; starting trees were Wagner trees with a random seed of 9999 replicates used with 10 trees save per replication. Our TNT analysis resulted in one most parsimonious tree of 317 steps. The consistency (CI) and retention indices (RI) are 0.57 and 0.78. Bremer support was assesses by using TNT software.

**Fig 14:**
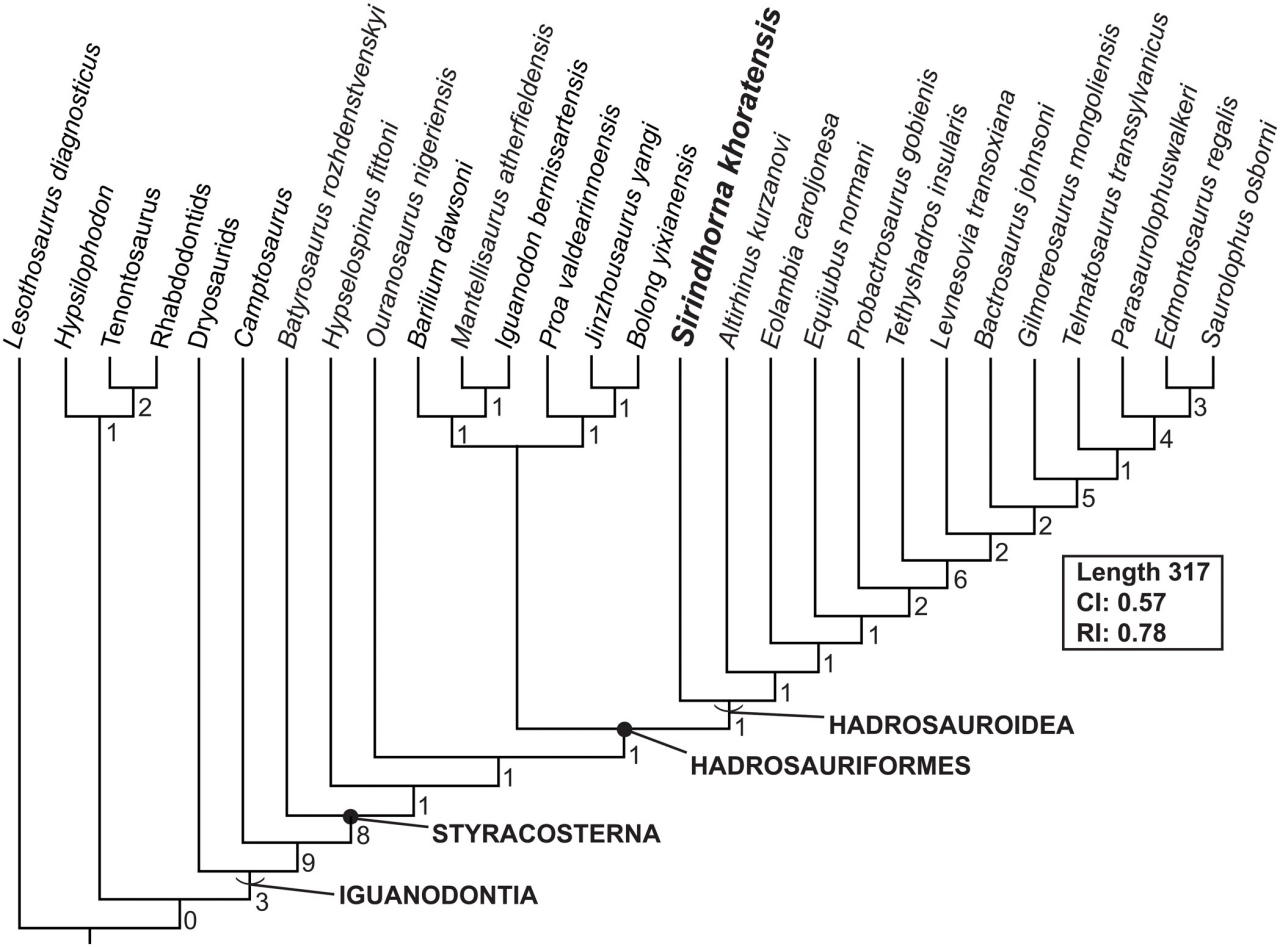
One MPT showing the phylogenetic position of *Sirindhorna khoratensis* based upon the data matrix of [37]. Definitions of the clade follow [37]. Bremer support indices are indicated on the right side of relevant branches.

Our tree recovers the same topology as that of [37], (Fig 14) with *Sirindhorna* settled as the sister taxon of (*Altirhinus* + more derived taxa). Therefore, *Sirindhorna* is the most basal hadrosauroid. Here, Hadrosauroidea is defined as all hadrosauriforms closer to *Saurolophus osborni* than to *Iguanodon bernissartensis*, and Hadrosuriformes is defined as the most recent common ancestor of *Saurolophus osborni* and *Iguanodon bernissartensis* and all of its decedents [28]. This position corresponds well with the result of above comparisons.

**Fig 15:**
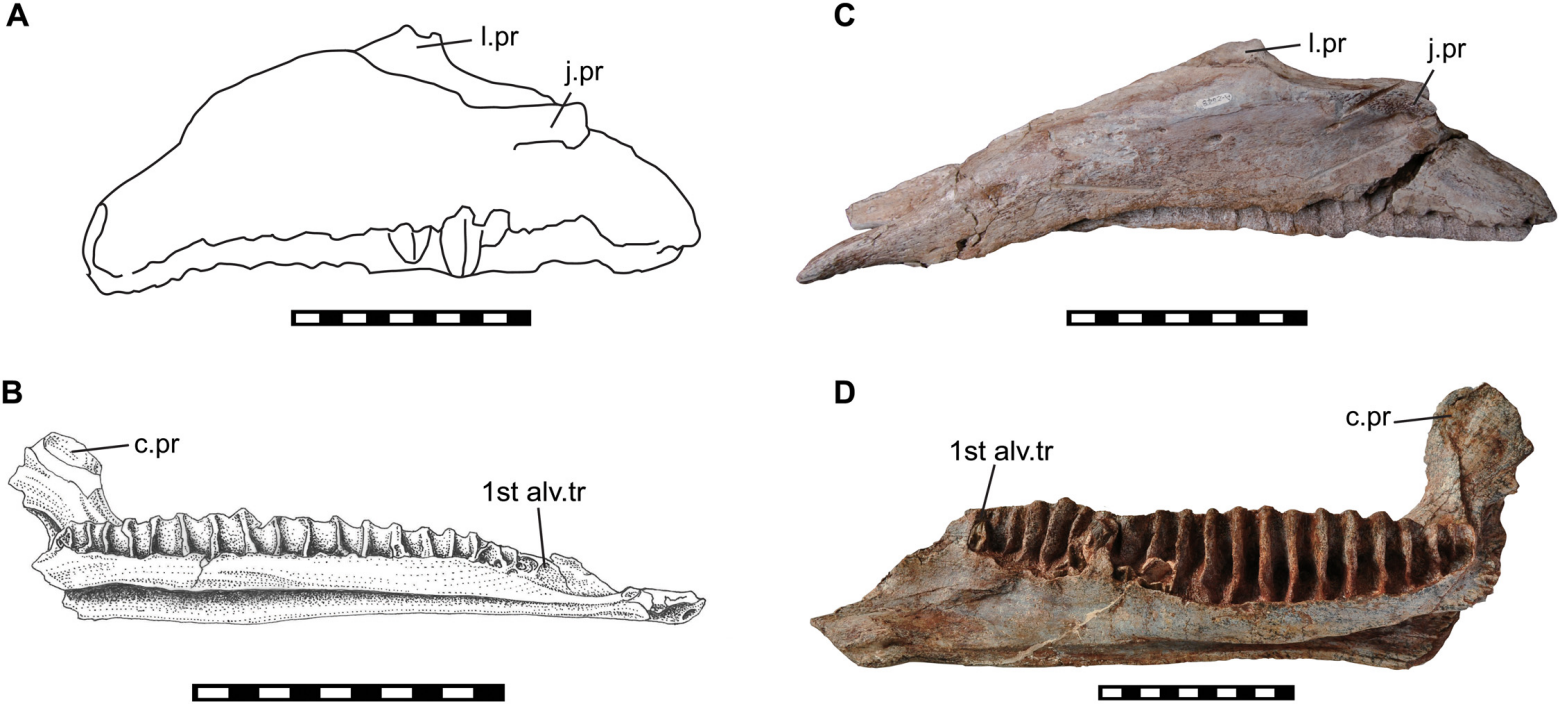
Comparisons with other Thailand iguanodontians. (A) Holotypic left maxilla of *Siamodon* (after [16]), (B) holotypic right dentary of *Ratchasimasaurus* (from [17]), (C) left maxilla of *Sirindhorna* (NRRU-A2048), (D) left dentary of *Sirindhorna* (NRRU3001-167). Scale bars equal 10 cm.

Two hadrosauroids have been previously known from the Khok Kruat Formation: *Siamodon nimingami* [16] and *Ratchasimasaurus suranareae* [17] (Fig 15). *Siamodon* was based upon an isolated maxilla. Although this maxilla was supposed to have been found in Ban Saphan Hin, one of the authors (PJ) insisted that the locality is Ban Nong Rangka, located in the adjacent subdistrict (Fig 1). The isosceles-shaped maxilla of *Siamodon* is distinct from the low triangle shape of that of *Sirindhorna* (Fig 15A and 15C). *Ratchasimasaurus*, on the other hand, is known only from one dentary, which is characterized with a low and elongate dentary ramus and the robust coronoid process. The morphology of *Ratchasimasaurs* is unique among iguanodontians and is evidently distinguishable from that of *Sirindhorna* (Fig 15B and 15D). Consequently, *Sirindhorna khoratensis* is regarded as a valid genus and species.

Hadrosauriforms were diversified in the Early Cretaceous of Asia, especially, from Barremian—Albian stages. For instance, *Fukuisaurus* and *Koshisaurus* from Japan, and *Bolong* and *Jinzhousaurus* from China were all found from Barremian—early Aptian stages. Later on, the Asian basal hadrosauroids are mainly discovered from Aptian-Albian stages: *Altirhinus* from Mongolia, *Jintasaurus*, *Xuwulong*, *Equijubus*, and *Gongpoquansaurus*, from Gansu Province and *Probactrosaurus* from Inner Mongolia, China. Now we have three Thailand hadrosauriforms from the Khok Kruat Formation (Aptian). Interestingly, as [23] mentioned, there is no iguanodontian records from the Cretaceous strata of Thailand in pre-and post-Khok Kruat formations. Dinosaur faunal changes between the underlying Sao Khua Formation (Barremian) and Khok Kruat Formation are remarkable [23]. Additional fossil records and geological investigations are needed to resolve dinosaur paleobiogeography in Thailand.

## Conclusions

**Fig 16:**
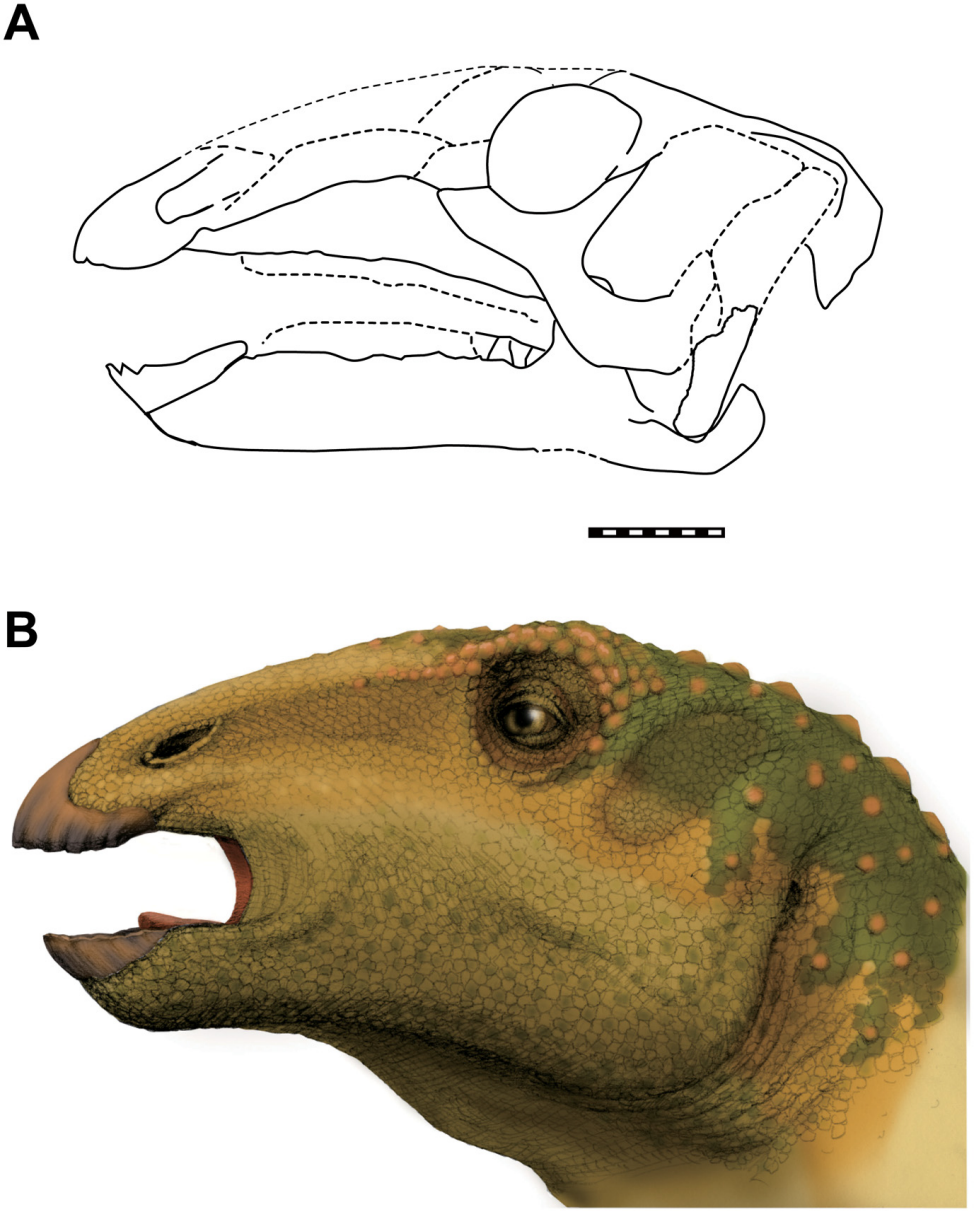
Skull of *Sirindhorna khoratensis*. (A) A composite skull reconstruction of *Sirindhorna*. Several elements are reversed. (B) Life restoration of the head of *Sirindhorna* by Yoko Ohnish. Scale bar equals 10 cm. Dashed line indicates missing elements.

The Early Cretaceous hadrosauroid dinosaur, *Sirindhorna khoratensis*, is described based upon cranial elements. This is the first report of well-preserved ornithopod skull in Southeast Asia (See reconstruction, in Fig 16). *Sirindhorna* shows general morphological features of hadrosauriforms, such as the low-triangle shaped maxilla, a broad leaf-shaped dentary tooth crown with one prominent primary and one secondary ridges, exclusion of the supraoccipital from the foramen magnum, and the closure of the antorbital fenestra. Uniquely, the craniocaudally-elongated parietals form a long saggital crest extending to the frontoparietal suture in *Sirindhorna*. Moreover, upper and lower jaws of *Sirindhorna* show evident differences from the other two Thailand hadrosauriforms, *Siamodon nimingami* and *Ratchasimasaurus suranareae*. Phylogenetic analysis recovers *Sirindhorna* as the most basal hadrosauroid.

## Supporting Information

S1 File The data matrix based on [37], including *Sirindhorna*. (TNT)

## Abbreviations

FPDM: Fukui Prefectural Dinosaur Museum, Fukui, Japan
IVPP: Institute of Vertebrate Paleontology and Paleoanthropology, Chinese Academy of Sciences, Beijing, China
NRRU: Northeastern Research Institute of Petrified Wood and Mineral Resources, Nakhon Ratchasima Rajabhat University, Nakhon Ratchasima, Thailand
SDSM: South Dakota School of Mines and Technology, Rapid City, South Dakota, USA
